# A novel DNA damage and repair‐related gene signature to improve predictive capacity of overall survival for patients with gliomas

**DOI:** 10.1111/jcmm.17406

**Published:** 2022-05-26

**Authors:** Xiaodong Li, Yichang Wang, Wei Wu, Jianyang Xiang, Maode Wang, Hai Yu

**Affiliations:** ^1^ Department of Neurosurgery The First Affiliated Hospital of Xi'an Jiaotong University Xi'an China; ^2^ Center of Brain Science The First Affiliated Hospital of Xi'an Jiaotong University Xi'an China

**Keywords:** gliomas, immunosuppressive status, prognostic prediction, risk stratification, the DDRRGs signature

## Abstract

Gliomas, as the most lethal and malignant brain tumours in adults, remain a major challenge worldwide. DNA damage and repair‐related genes (DDRRGs) appear to play a significant role in gliomas, but the studies of DDRRGs are still insufficient. Herein, we systematically explored and analysed 1547 DDRRGs in 938 glioma samples from TCGA and CGGA datasets. Using least absolute shrinkage and selection operator (LASSO) Cox regression analysis, we identified a 16‐DDRRG signature, characterized by high‐risk and low‐risk patterns. This risk model harbours robust predictive capability for overall survival of glioma patients. We found the high‐risk score is strongly associated with well‐known malignant features of gliomas, such as the mesenchymal subtype, IDH‐wildtype, 1p/19q non‐codeletion and MGMT promoter unmethylated status. In addition, we found that the high‐risk score is also linked with multiple oncogenic pathways and therapeutic resistance. Significantly, we found the high‐risk group has higher enrichment of immunosuppressive cells (M2‐type macrophages, Tregs and MDSCs) and immune inhibition biomarkers (PD‐1, PD‐L1 and CTLA‐4). Lastly, we proved that SMC4, which has the highest positive regression coefficient in our risk model, is strongly linked with malignant progression and TMZ resistance of gliomas in a E2F1‐dependent manner.

## 1 INTRODUCTION

Gliomas are the most common primary tumours in the central nervous system in adults, accounting for approximately 80% of primary malignant and 26.6% of all brain tumours.^1^ The characteristics of gliomas are composed by highly invasive and proliferative features, widespread heterogeneity, therapeutic resistance and inexorable recurrence.^2^, ^3^ Currently, glioma patients are generally treated with maximum surgical resection, chemotherapy (temozolomide, TMZ) and irradiation (IR) therapy.^4^ However, the survival time for patients with gliomas has no significant improvement.^5^ A study has showed that patients with lower‐grade gliomas (LGGs) have heterogenous overall survival (OS), from 1 year to 15 years,^6^ while the average survival time of GBM is less than 20 months after diagnosis.^5^

In 2016, the World Health Organization (WHO) classification of gliomas used molecular parameters including isocitrate dehydrogenase 1 and 2 (IDH1 and IDH2) mutations and 1p/19q codeletion status in addition to histopathological criteria.^7^ In addition, a study indicated that methylation status of the O^6^‐methylguanine‐DNA methyltransferase (MGMT) promoter is a molecular biomarker for chemotherapy.^8^ In 2010, GBM was divided into proneural, neural, classical and mesenchymal subtypes,^9^ but recent study has not classified neural as major subtype due to lack of tumour‐intrinsic patterns.^10^ However, existing molecular classification has not led to improvement of outcomes for glioma patients.^11^ Therefore, more comprehensive studies are urgently needed to provide predictive models for gliomas.

DNA damage response (DDR) is the pathway that cells recognize and repair DNA damage, which is required to maintain the genomic integrity and stability.^12^ Increasing studies found that DNA damage induced by chemicals and physical agents can promote carcinogenic mutations that led to human cancers.^13^ Meanwhile, accumulating evidence showed deficiency in DDR facilitates progression of multiple cancers, such as brain metastases of colorectal cancer^14^ and pancreatic cancer.^15^ Interestingly, studies also showed that the overactivated DDR can induce the therapy resistance of glioma stem cells, and targeting DDR pathway can overcome this resistance.^16^ Consistently, a report suggested that Rad51, a DNA double‐strand break repair gene, accounts for resistance to DNA‐damaging reagents such as chemotherapy.^17^ In addition, a study showed that purine metabolites could induce radioresistance by enhancing the repair of DNA double‐stranded breaks.^18^ Another study also reported that p53, the key molecule of DDR, can regulate M2 polarization of microglia to remodel immunosuppressive microenvironment of gliomas.^19^ Thus, the DDR is a potent candidate for prognosis prediction in patients with gliomas. Previous studies have identified a variety of DNA damage and repair‐related genes that are involved in the DDR process, providing additional potential choices for therapeutic strategies.^20^ Thus, our studies focused on the role of DDRRGs in gliomas.

In this study, we explored DDRRGs features from the Cancer Genome Atlas (TCGA) and the Chinese Glioma Genome Atlas (CGGA) datasets. The results indicated that patients with gliomas can be divided into 2 clusters with distinct gene features and clinical outcome based on consensus clustering. Next, the DDRRG signature was established using least absolute shrinkage and selection operator (LASSO) Cox regression analysis. We found that the DDRRG signature, composed of FBXO18, MMS19, SMC4, HEXB, UBQLN4, VAV3, E2F7, EFNB1, WEE1, SAA1, SHISA5, WAC, PSMC2, PTGFRN, EIF3L and HMGA2, can independently predict the outcome of glioma patients. Moreover, we found that this risk signature is strongly linked with multiple oncogenic pathways, immunosuppressive tumour microenvironment and therapeutic response. In addition to bioinformatics analyses, we functionally confirmed the oncogenic role of SMC4 in gliomas in vivo and in vitro.

## 2 MATERIALS AND METHODS

### 2.1 Included patients and datasets

In total, 938 glioma samples from two cohorts have been involved in this study. The data of mRNA expression was downloaded from TCGA RNA sequencing (RNA‐seq) dataset^21^ and CGGA RNA‐seq dataset.^22^, ^23^ Detailed information was provided in the Data S1. The corresponding clinical information was obtained from TCGA dataset (https://portal.gdc.cancer.gov/) and CGGA dataset (http://www.cgga.org.cn), respectively. The clinicopathological features for 938 patients were shown in Tables 1 and S1. For TCGA dataset, appropriate consents were obtained from relevant institutional review boards, which coordinated the consent process at each tissue‐source site. For the CGGA dataset, written informed consents were obtained from all patients.^22^, ^23^

**TABLE 1:** Clinical feature of patients in cluster 1 and cluster 2 in TCGA dataset

Characteristics	*N*	Cluster 1	Cluster 2	*p* Value
Total cases	629	239	390	
Gender				
Male	329	141	188	0.077
Female	242	86	156	
Age (years)				
≤47	289	51	238	<0.001
>47	282	176	106	
Grade				
II	210	9	201	<0.001
III	228	73	155	
IV	144	143	1	
Subtype				
Classical	81	81	0	<0.001
Mesenchymal	90	78	12	
Proneural	223	34	189	
Neural	104	15	89	
IDH status				
Mutation	404	31	373	<0.001
Wildtype	218	204	14	
MGMT promoter				
Methylation	450	96	354	<0.001
Unmethylation	149	113	36	
1p19q				
Codel	157	1	156	<0.001
Non‐codel	466	232	234	

### 2.2 The construction of risk signature

**FIGURE 1:**
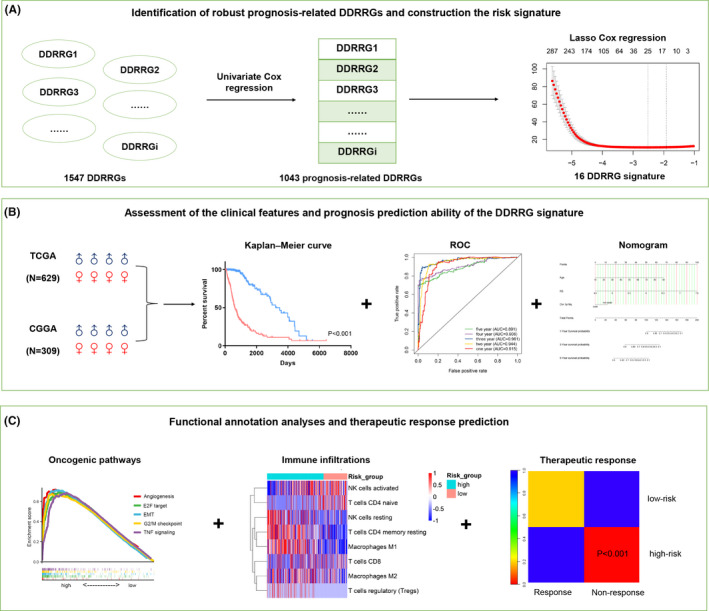
Main schematic workflow for analysing DNA damage and repair‐related genes (DDRRGs) in gliomas. (A) Identification of the most robust prognosis‐related DDRRGs in gliomas and construction of DDRRG risk model using the univariate Cox regression and least absolute shrinkage and selection operator (LASSO) regression analyses. (B) Assessment of the clinical features and prognosis prediction ability of the DDRRG signature. (C) Functional annotation analyses and evaluation of therapeutic response difference between high‐risk and low‐risk groups

Comprehensive analysis was performed using TCGA and CGGA datasets to identify and construct a prognosis‐related gene signature that captured DDRRGs, as shown in Figure 1, hereafter referred to as the DDRRG signature. Glioma samples including detailed survival information were used in this process. The list of DDRRGs was obtained from GSEA gene sets (https://www.gsea‐msigdb.org/gsea/index.jsp) by using ‘DNA and damage’ or ‘DNA and repair’ as the keyword. At last, 1547 DDRRGs were included in the study. In TCGA and CGGA datasets, univariate Cox regression analysis was performed to analyse the predictive value of 1547 DDRRGs. In total, 1043 DDRRGs were identified to be associated with glioma prognosis in both datasets.

Subsequently, the 1043 DDRRGs were analysed by LASSO COX regression analysis^24^ to select out the most robust prognostic genes. Using the R package (‘glmnet’, 4.1.1), we performed the LASSO COX regression model to minimize the over‐fitting and identify the most powerful prognosis‐associated DDRRGs in gliomas using 10‐fold cross‐validation. At last, 16 DDRRGs were identified to construct the DDRRGs signature. The DDRRG signature = (−0.0579 × Exp_FBXO18) + (−0.0029 × Exp_MMS19) + (0.2184 × Exp_SMC4) + (0.0478 × Exp_HEXB) + (−0.0036 × Exp_UBQLN4) + (0.0066 × Exp_VAV3) + (0.0149 × Exp_E2F7) + (0.0134 × Exp_EFNB1) + (0.0875 × Exp_WEE1) + (0.0391 × Exp_SAA1) + (0.1342 × Exp_SHISA5) + (−0.0122 × Exp_WAC) + (0.1369 × Exp_PSMC2) + (0.0707 × Exp_PTGFRN) + (−0.0492 × Exp_EIF3L) + (0.0272 × Exp_HMGA2). (Exp represents the expression level of each selected gene). (Table S2). Based on this formula, the risk score (RS) for each sample was calculated in TCGA and CGGA datasets, and the median value was manually defined as the threshold for high‐risk and low‐risk. Time‐dependent ROC analysis was performed to verify the predictive accuracy of the risk signature.

### 2.3 Bioinformatics analyses and experiments in vitro and in vivo

The details for bioinformatics analyses and experiments in vitro and in vivo in this study were described in the Data S1.

### 2.4 Statistics

The statistical analyses were performed by using the R software (version 4.0.3, details for related packages were described in the Data S1), Prism 6 (GraphPad Software, K‐M survival analysis) and SPSS (version 22.0, chi‐square test and univariate and multivariate Cox regression analyses). The role of each analysis in this study was provided in the Data S1. *p* < 0.05 was considered as statistical significance.

## 3 RESULTS

### 3.1 Using the DDRRGs for the consensus clustering of gliomas

To explore the potential oncogenic role of DDRRGs in gliomas, consensus clustering method was applied to analyse in TCGA and CGGA datasets. Cumulative distribution function (CDF) was carried out to determine the optimum cluster number. The results showed that clustering outcoming is stable when k = 2 (Figures S1A and S2A–C). Heatmaps showed distinct DDRRGs distribution between cluster 1 and cluster 2 (Figures S1B and S2D, Table S3). The K‐M curve showed that the OS of cluster 1 is apparently poorer than that of cluster 2 (Figures S1C and S2E). To explore the clinical relevance of the clusters, we calculated the proportion of patients with different clinical characteristics in each cluster. The results were shown by percent stacked column charts (Figures S1D and S2F). Meanwhile, chi‐square test was performed. The results showed that patients in cluster 1 have more malignant features, such as the mesenchymal subtype, IDH‐wildtype status, 1p/19q non‐codeletion and non‐methylation of MGMT promoter, while the cluster 2 had absolutely opposite clinical patterns (Table 1). Consistently, we found the similar distribution pattern of clinical features in the CGGA dataset (Table S1). These results indicate that DDRRGs are strongly associated with molecular features and clinical outcome for patients with gliomas.

### 3.2 Exploration of the DDRRG signature in gliomas

**FIGURE 2:**
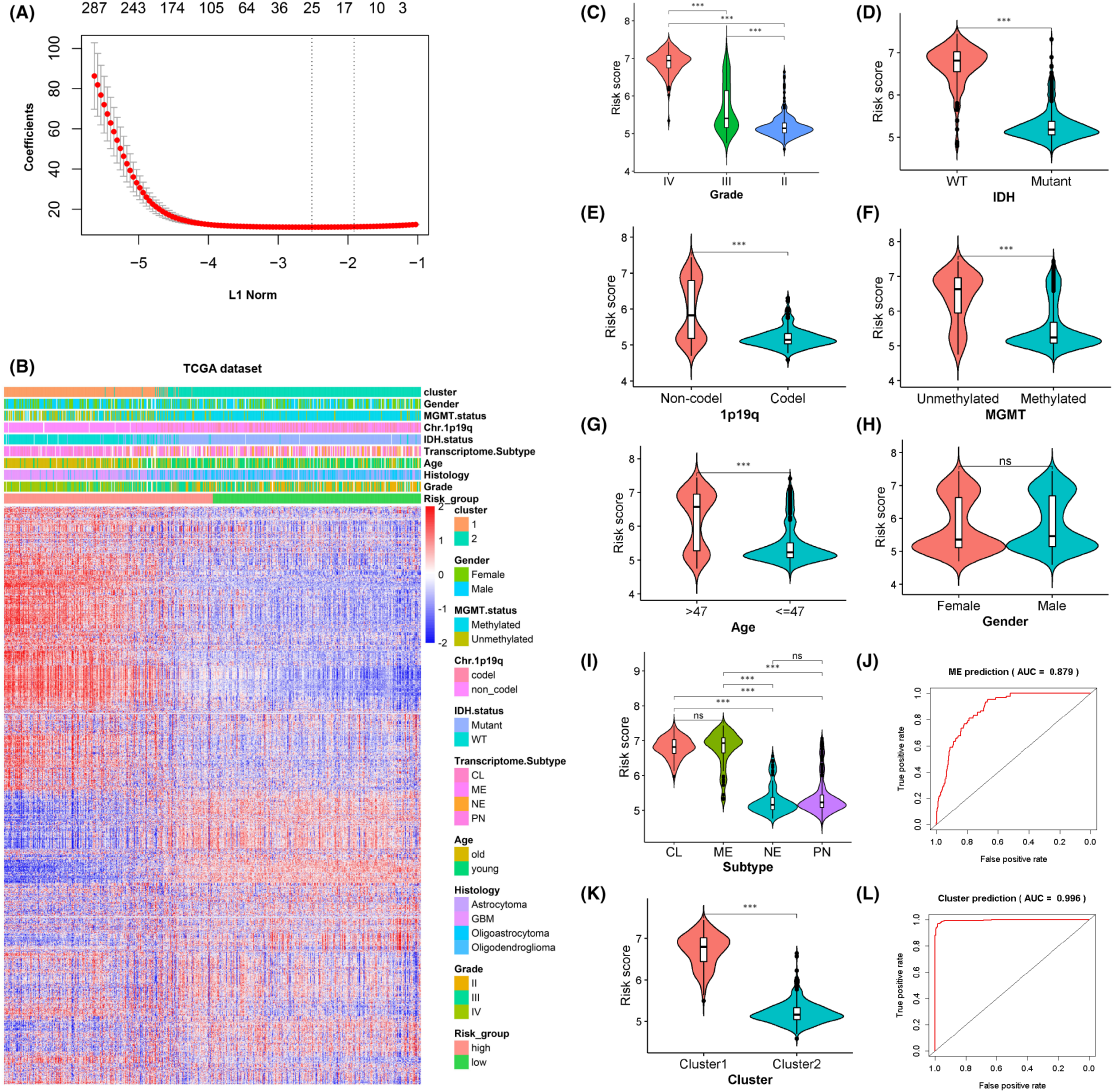
Exploration of the clinical characteristics of the DNA damage and repair‐related genes (DDRRGs) signature in gliomas in TCGA dataset. (A) Cross‐validation for tuning parameter selection using the least absolute shrinkage and selection operator (LASSO) regression analysis. (B) The heatmap showing the expression features of 1547 DDRRGs and corresponding clinical patterns. (C–I) Violin plots comparing the risk score for patients with gliomas sub‐grouped by WHO grade, IDH mutation status, 1p/19q status, MGMT promoter methylation status, age, gender or subtypes. (J) ROC curve showing the predictive ability of the DDRRG signature for the mesenchymal subtype. (K) Violin plot comparing the risk score for patients with gliomas between cluster 1 and cluster 2. (L) ROC curve showing the predictive ability of the risk model for clusters. ***p < 0.001, ns, not significant

Now that DDRRGs had distinct cluster patterns in gliomas, we decided to construct a model that could predict this feature. Firstly, we identified 1043 prognostic‐related DDRRGs using univariate Cox regression analysis as described in the method section. Next, LASSO Cox regression model was used to identify the most robust predictive genes (non‐zero coefficients). In total, 16 genes were identified, including FBXO18, MMS19, SMC4, HEXB, UBQLN4, VAV3, E2F7, EFNB1, WEE1, SAA1, SHISA5, WAC, PSMC2, PTGFRN, EIF3L and HMGA2 (Figures 2A and S3A). Consequently, the 16 gene‐related score was calculated based on the formula mentioned in the Materials and Methods section in TCGA and CGGA datasets, respectively. Based on the median value of risk scores, patients with gliomas were classified into high‐risk and low‐risk groups. The heatmaps showed different gene expression level and clinical patterns between high‐ and low‐risk groups (Figures 2B and S3B). Further analysis in TCGA dataset suggested that the risk score is positively correlated with WHO grade of gliomas (Figure 2C). In addition, patients with IDH‐wildtype status, 1p/19q non‐codeletion, non‐methylation of MGMT promoter or higher age had elevated risk score (Figures 2D–G). However, we observed no statistical difference between male and female groups in TCGA dataset (Figure 2H). Additionally, the results suggested that the classical and mesenchymal subtypes have highest risk score (Figure 2I). ROC analysis was performed to evaluate the predictive capability of risk model for the mesenchymal subtype. The results revealed that the area under curve (AUC) is 87.9% in predicting the mesenchymal subtype in TCGA dataset (Figure 2J). Next, we assessed whether the risk signature matches the identified cluster. The results indicated that glioma patients in cluster1 have higher risk score (Figure 2K). Consistently, the AUC in predicting the cluster was 99.6% in TCGA dataset (Figure 2L). Same analyses were performed in the CGGA dataset, and we obtained similar results (Figure S3C–J). These results imply that the high‐risk score may be linked with aggressive progression in gliomas.

### 3.3 The prognostic value of the DDRRG signature

**FIGURE 3:**
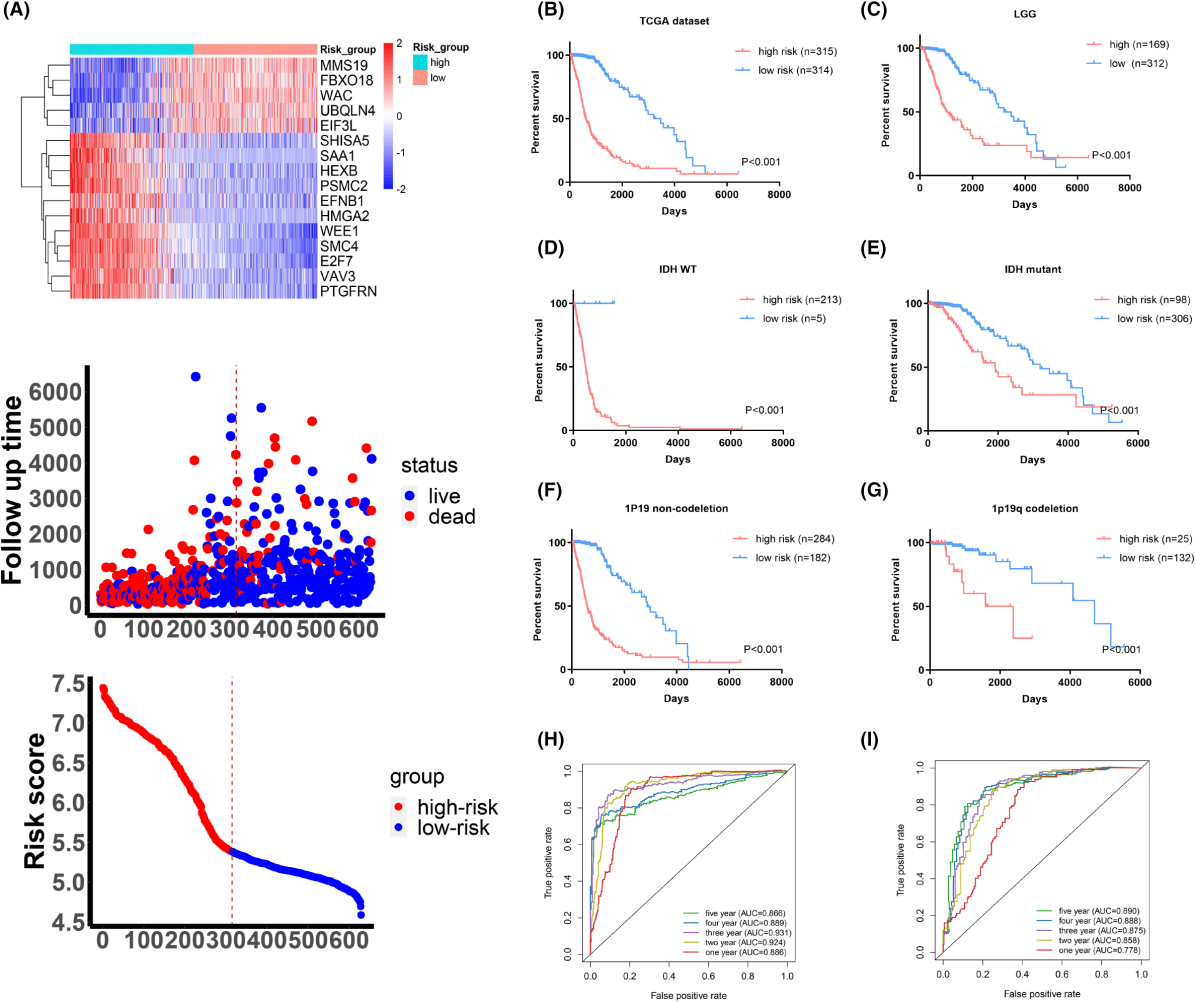
Prognostic value of the DNA damage and repair‐related genes (DDRRGs) model in TCGA dataset. (A) Distribution of the risk score, expression level of 16 genes and prognostic status between high‐risk and low‐risk groups. (B–G) K‐M curves comparing prognosis for patients with gliomas sub‐grouped by grade, IDH mutation or 1p/19q status between high‐risk and low‐risk groups. (H,I) ROC curves showing the satisfactory predictive value of the risk signature for 1‐, 2‐, 3‐,4‐ and 5‐year prognosis in TCGA and CGGA datasets

To comprehensively understand the prognosis prediction ability of the DDRRG signature, we evaluated the association between expression level of DDRRGs, risk score and patients' prognosis. The results suggested that patients in the high‐risk group have higher expression of SMC4, HEXB, VAV3, E2F7, EFNB1, WEE1, SAA1, SHISA5, PSMC2, PTGFRN and HMGA2, and suffer worse outcome. Contrarily, the patients in the low‐risk group have higher expression of FBXO18, MMS19, UBQLN4 and WAC, and have better outcome (Figures 3A and S4A). Meanwhile, K‐M survival analysis indicated that these 16 genes can distinguish the prognosis of patients independently (Figures S5 and S6). In addition, K‐M curves showed that patients in the high‐risk group suffer worse prognosis (Figures 3B and S4B). To further explore the relationship between OS and risk groups, we performed K‐M survival analysis in each subgroup of gliomas. The results showed that patients sub‐grouped by tumour grade, IDH mutation, 1p/19q codeletion or MGMT promoter unmethylated status, suffer worse outcome in the high‐risk group in TCGA dataset (Figures 3C–G and S7A). Meanwhile, the same analyses from the CGGA dataset validated these results (Figures S4C–H and S7B). Additionally, K‐M survival analysis suggested that our risk signature has satisfactory value in predicting OS of patients stratified by age or gender (Figure S7C–D). Subsequently, univariate and multivariate Cox regression analyses confirmed that the risk score is an independent prognosis factor for gliomas (Tables 2 and S4). Next, we performed ROC analysis to evaluate the predictive value of this risk model in predicting outcome of patients. The results showed that our risk signature harbours high AUC values for TCGA dataset (1‐year: 88.6%, 2‐year: 92.4%, 3‐year: 93.1%, 4‐year: 88.9% and 5‐year: 86.6%) (Figure 3H). Meanwhile, the results from CGGA dataset also showed satisfactory AUC values (1‐year:77.8%, 2‐year: 85.8%, 3‐year: 87.5%, 4‐year: 88.8% and 5‐year: 89.0%) (Figure 3I). These results suggest that our risk signature has robust predictive value for patients' OS.

**TABLE 2:** Univariate and multivariate analyses of risk score and clinical features in TCGA dataset

Variables	Univariate analysis		Multivariate analysis	
	HR (95% CI)	*p* Value	HR (95% CI)	*p* Value
Risk score	7.100 (5.579–9.036)	<0.001	4.572 (2.762–7.569)	<0.001
Age	1.071 (1.059–1.083)	<0.001	1.041 (1.027–1.055)	<0.001
Gender	0.899 (0.658–1.229)	0.427		
WHO Grade				
III	3.055 (1.987–4.697)	<0.001	1.275 (0.780–2.083)	0.332
IV	21.520 (13.491–34.329)	<0.001	1.202 (0.606–2.382)	0.598
IDH status	0.095 (0.069–0.131)	<0.001	1.229 (0.641–2.357)	0.535
MGMT status	0.317 (0.234–0.427)	<0.001	0.870 (0.616–1.227)	0.427
1p19q status	0.237 (0.147–0.382)	<0.001	0.564 (0.323–0.985)	0.044

### 3.4 Construction a prediction model based on clinical features

**FIGURE 4:**
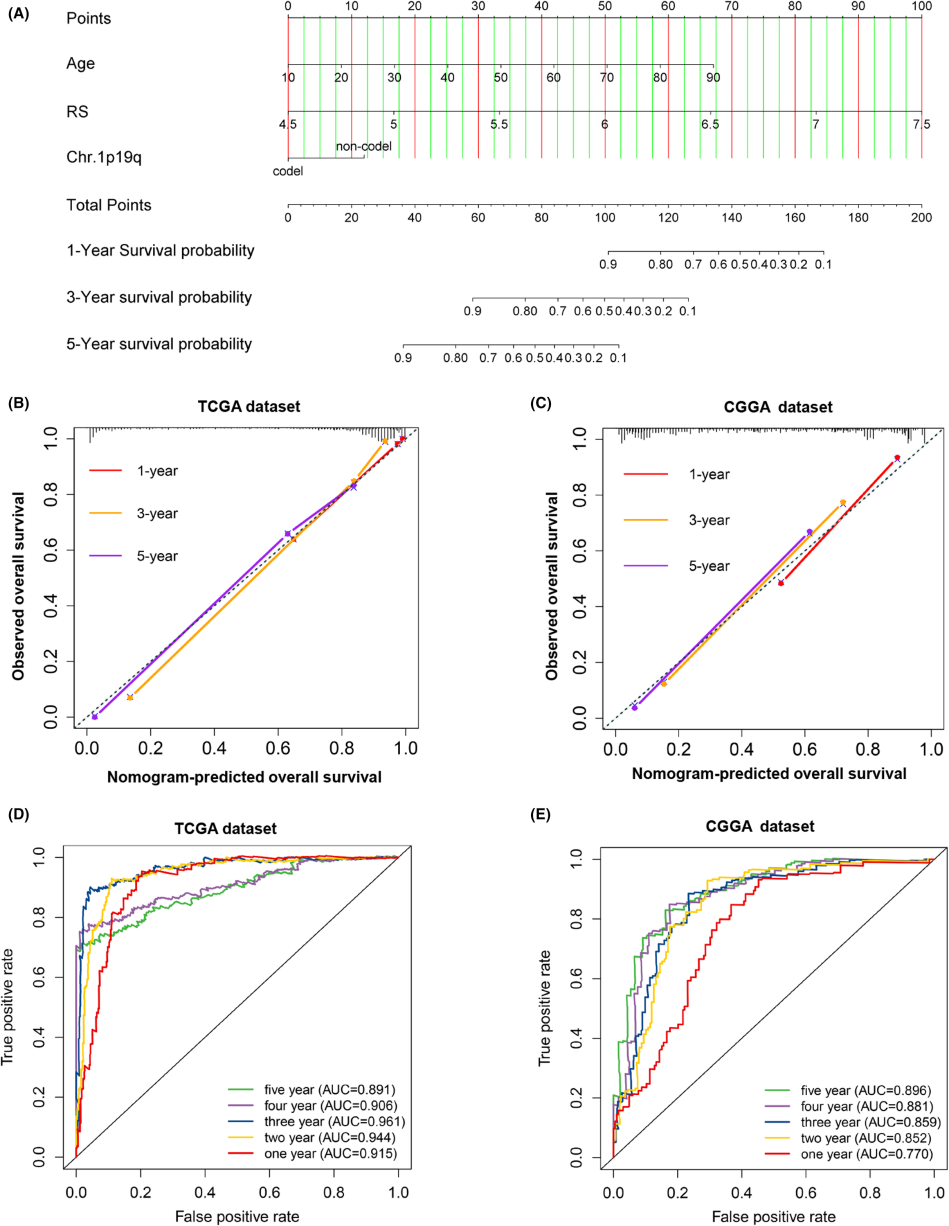
Independent prediction model integrating clinical information for prognosis of glioma patients. (A) The nomogram predicting 1‐, 3‐ and 5‐year prognosis in patients with gliomas in TCGA dataset. (B, C) Calibration plots predicting the accuracy of nomogram at 1‐, 3‐ and 5‐year in TCGA and CGGA datasets. (D, E) ROC curves showing the predictive value of the scores from nomogram model for 1‐, 2‐, 3‐,4‐ and 5‐year prognosis in TCGA and CGGA datasets

To further explore the value of applying our risk model to the clinical, we integrated the risk score and independent clinical parameters of patients with gliomas using a nomogram model. The C‐indexes were 0.883 (TCGA) and 0.764 (CGGA), suggesting the satisfactory value of our signature (Figure 4A). In addition, the calibration curves manifested a favourable consistence between observational and predictive values for patients' OS (Figure 4B,C). Lastly, we calculated scores of nomogram model and performed ROC analysis based on these scores. The results indicated that the model after integrating clinical information has apparently improved AUC values for TCGA dataset (1‐year: 91.5%, 2‐year: 94.4%, 3‐year: 96.1%, 4‐year: 90.6% and 5‐year: 89.1%) (Figure 4D). Meanwhile, integrated clinical model still had high AUC values for the CGGA dataset (1‐year: 77.0%, 2‐year: 85.2%, 3‐year: 85.9%, 4‐year: 88.1% and 5‐year: 89.6%) (Figure 4E). These results validate the value of clinical application of our risk signature.

### 3.5 Functional annotation of the risk signature

**FIGURE 5:**
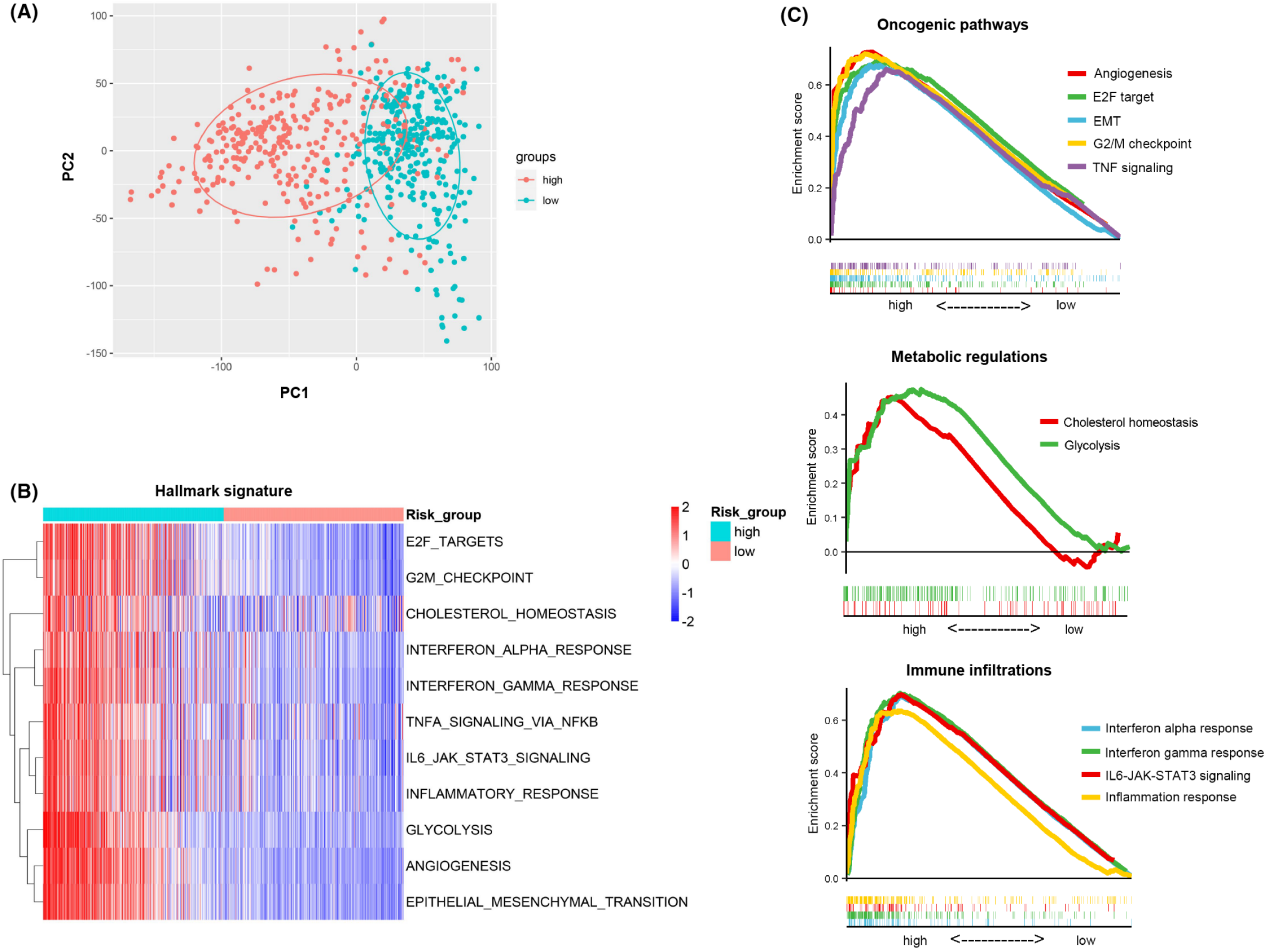
Functional analyses of the DNA damage and repair‐related genes (DDRRGs) signature in TCGA dataset. (A) PCA analysis showing distinct gene expression distribution between high‐ and low‐risk groups. (B) GSVA analysis was performed to evaluate meta‐score of hallmark signature in each sample of gliomas in TCGA dataset. The heatmap showing meta‐score of each hallmark signature between high‐risk and low‐risk groups. (C) GSEA analyses revealing the highly enriched oncogenic pathways, metabolic alternations and immune infiltrations in the high‐risk group

Deregulation of oncogenic pathways are considered to be tightly linked with clinical therapeutic response. Thus, we next further characterized the functional differences between the high‐risk and low‐risk groups. Firstly, principal component analysis (PCA) was carried out, and the results showed significantly different transcriptional expression patterns of DDRRGs between high‐ and low‐risk groups in both datasets (Figures 5A and S8A). Next, gene set variation analysis (GSVA) was performed to explore the pathways in high‐risk and low‐risk groups. The heatmaps presented obtained meta‐score from GSVA (Figures 5B and S8B). The results suggested that the high‐risk group is positively linked with oncogenic pathways related to gliomas that have been proved before, such as angiogenesis,^5^ E2F target,^25^ epithelial‐mesenchymal transition (EMT),^26^ G2/M checkpoint^27^ and tumour necrosis factor‐transcription factor nuclear factor kappa B (TNF‐NFκB) signalling.^28^ In addition, we also found that metabolic alternations (glycolysis and cholesterol homeostasis) and immune regulations (interferon alpha response, interferon gamma response, IL6‐JAK‐STAT3 signalling and inflammation response) are also enriched in the high‐risk group. In addition, we performed GSEA to further validate our results. Consistently, we obtained similar results via GSEA analyses (Figures 5C and S8C).

The risk model was constructed based on DNA damage and repair‐related genes. We therefore specifically investigated the DNA repair‐related pathways. Current evidence showed that 5 main types are involved in DNA repair in GBM, including homologous recombination repair (HRR), non‐homologous end‐joining (NHEJ) pathways, base excision repair (BER), nucleotide excision repair (NER) and mismatch repair (MMR).^29^ Therefore, we performed GSEA analysis and found that HRR, MMR and BER are enriched in the high‐risk group in both datasets (Figures S9A,B).

Given the above findings that glycolysis and cholesterol homeostasis are enriched in high‐risk group, we further explored the association of our signature DDRRGs in regulation of metabolic alternations based on 114 metabolic pathways obtained from the previous study.^30^ GSVA was performed to compute the meta‐score of each patient for metabolic pathways. The heatmaps showed 10 glioma‐related metabolic pathways that are enriched in the high‐risk group (Figure S9C,E). In addition, we observed that several pathways that were reported to be tightly linked with malignant behaviour for gliomas have elevated enrichment in the high‐risk group, such as pyrimidine metabolism^31^ and purine metabolism^18^ (Figure S9D,F). These above results suggest that our risk signature is tightly associated with malignant progression of gliomas.

### 3.6 High‐risk score is closely linked with immunosuppressive microenvironment of gliomas

**FIGURE 6:**
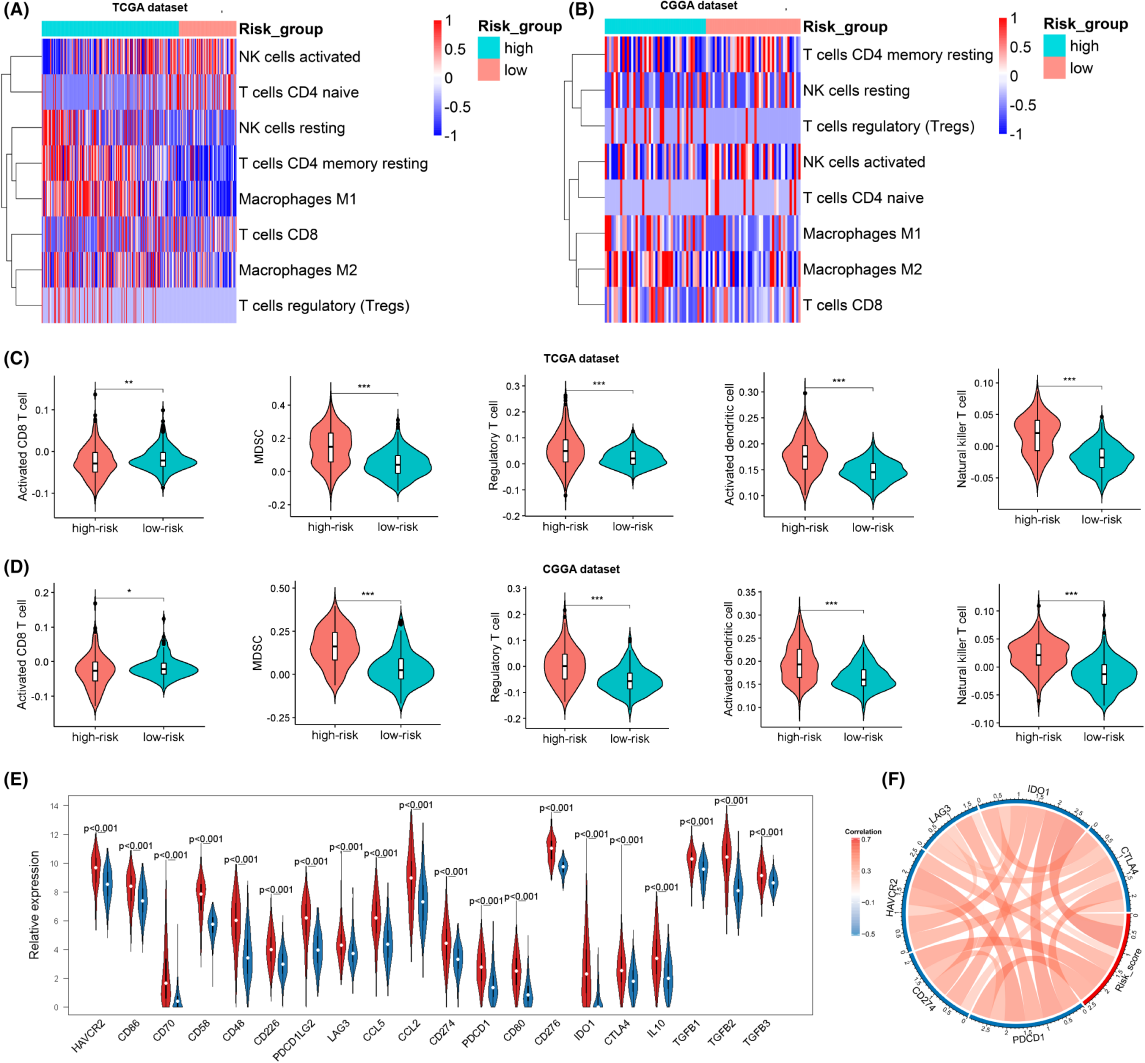
High‐risk score is closely linked with immunosuppressive microenvironment of gliomas. (A, B) The heatmaps showing expression of selected immune cell subpopulations using CIBERSORT algorithms between high‐risk and low‐risk groups in TCGA and CGGA datasets. (C, D) Violin plots comparing meta‐score of immune cells between high‐risk and low‐risk groups in TCGA and CGGA datasets. (E) Comparison analyses showing expression level of immunosuppressive biomarkers between high‐risk and low‐risk groups in TCGA dataset. (F) CIRCOS plot showing the relationship between immune checkpoint markers and the risk score in TCGA dataset. *p < 0.05, **p < 0.01, ***p < 0.001

Given the promising role of immune therapy in glioma treatment^32^ and the significant immune alternation difference between the risk groups, we carried out further investigation on this aspect. We firstly calculated absolute proportion of 22 tumour infiltrating immune cells (TIICs) for each glioma sample using deconvolution algorithms of CIBERSORT. The heatmap was used to show the distinct expression of selected immune cell subpopulations between high‐risk and low‐risk groups. The results showed that M2‐type macrophages have highest expression in the high‐risk group in both datasets. Interestingly, previous evidence has proved that M2‐type macrophages can promote immunosuppressive microenvironment and progression of gliomas.^33^ In addition, we found that T cell regulatory (Tregs) have higher expression in the high‐risk group (Figure 6A,B). To verify the above results and explore the expression of CD8 T cells subpopulations between high‐risk and low‐risk groups, we performed ssGSEA algorithm to calculate the abundance of 28 immune‐related cell types. The heatmap visualized the results (Figure S10A–D). We observed that activated dendritic cells, myeloid‐derived suppressor cells (MDSCs), natural killer T cells and Tregs were highly elevated in the high‐risk group. Meanwhile, the results showed that activated CD8 T cell was negatively linked with RS (Figure 6C–D). Next, we performed correlation analyses and further validated these results (Figure S10C–D). Previous studies have well demonstrated that MDSCs and Tregs promoted the formation of immunosuppressive microenvironment.^34^ Therefore, we inferred that glioma patients in high‐risk group may have an immunosuppressive microenvironment. To validate our hypothesis, we compared expression of immune suppressive biomarkers, including secreted immune inhibitory factors and immune checkpoint markers, between high‐ and low‐risk groups. Comparison analysis revealed that the selected immune inhibitory biomarkers all have higher expression in the high‐risk group compared with that in the low‐risk group (Figures 6E and S10E). Correlation analysis further validated that immune checkpoint biomarkers, such as PDCD1 (PD‐1), CD274 (PD‐L1), HAVCR2 (TIM3), LAG3, IDO1 and CTLA‐4, were highly expressed in the high‐risk group (Figures 6F and S10F). These analyses indicate that high‐risk score is strongly linked with immunosuppressive role for gliomas.

### 3.7 High‐risk score is potentially associated with therapy resistance in gliomas

**FIGURE 7:**
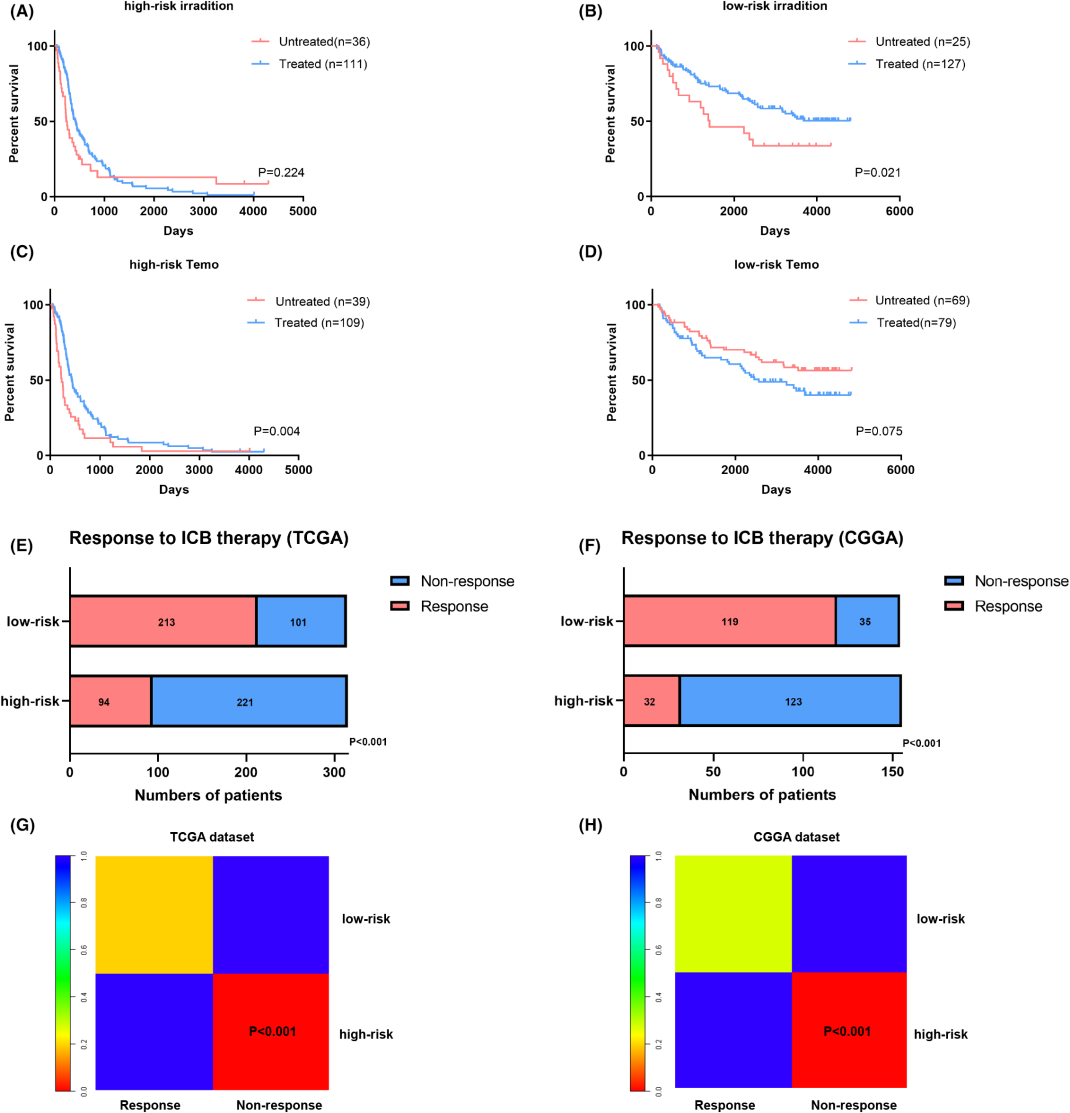
High‐risk score is potentially associated with therapy resistance in gliomas. (A, B) K‐M curves showing OS for glioma patients in the high‐risk group or low‐risk group that treated with or without irradiation. (C, D) K‐M curves showing OS for glioma patients in the high‐risk group or low‐risk group that treated with or without TMZ. (E, F) Stacked column charts comparing the response of gliomas to ICB therapy between high‐ and low‐risk groups in TCGA and CGGA datasets using TIDE dataset. (G, H) The response of gliomas to ICB therapy between high‐ and low‐risk groups in TCGA and CGGA datasets using expression data of GSE78220

Based on aforementioned results, we next explored the association between the risk model and therapeutic response in gliomas using clinical information from the CGGA dataset. K‐M survival analysis was performed to compare prognosis of glioma patients treated with or without irradiation/TMZ. The results showed no statistical difference between untreated and treated groups for irradiation therapy in the high‐risk group (Figure 7A), while we observed that untreated group suffered worse outcome for irradiation therapy in the low‐risk group (Figure 7B), suggesting that high‐risk score was positively linked with radioresistance. However, there was no statistical difference between untreated and treated group for TMZ therapy in the high‐risk or low‐risk groups (Figure 7C,D).

The above results showed that high‐risk score is likely linked with immunosuppressive tumour microenvironment. Thus, in addition to conventionally therapy, we next decided to explore the relationship between risk score and immunotherapy responsiveness using online TIDE database. The results revealed that the high‐risk score was indicative for a resistant phenotype to ICB therapy in gliomas (Figure 7E,F). In addition, we used subclass mapping algorithm to further validate the findings in TIDE. The expression data of 28 patients that underwent anti‐PD‐1 therapy was obtained from previous study.^35^ The results showed that glioma samples with high‐risk score are more potentially resistant to ICB therapy (Figure 7G,H).

Accumulating evidence indicated that mutational load is closely linked with immunotherapy.^35^, ^36^ Interestingly, previous study showed that DNA damage was strongly linked with carcinogenic mutations.^37^ Therefore, we evaluated the variation of somatic mutation between high‐ and low‐risk groups in our risk model. The results revealed that high‐risk group is marked by malignant biomarkers, such as TP53 mutation (38%), EGFR mutation (20%) and PTEN mutation (20%)^38^ (Figure S11A). In addition, we observed that IDH1 mutation (92%) indicating better OS of patients^38^ was the feature of low‐risk group (Figure S11B). Subsequently, TMB of each patient was calculated and the results indicated that high‐risk sore has significantly higher TMB (Figure S11C). Above results indicated that high‐risk score is a phenotype of therapy resistance.

### 3.8 Functional verification of carcinogenic effect of SMC4 in gliomas

**FIGURE 8:**
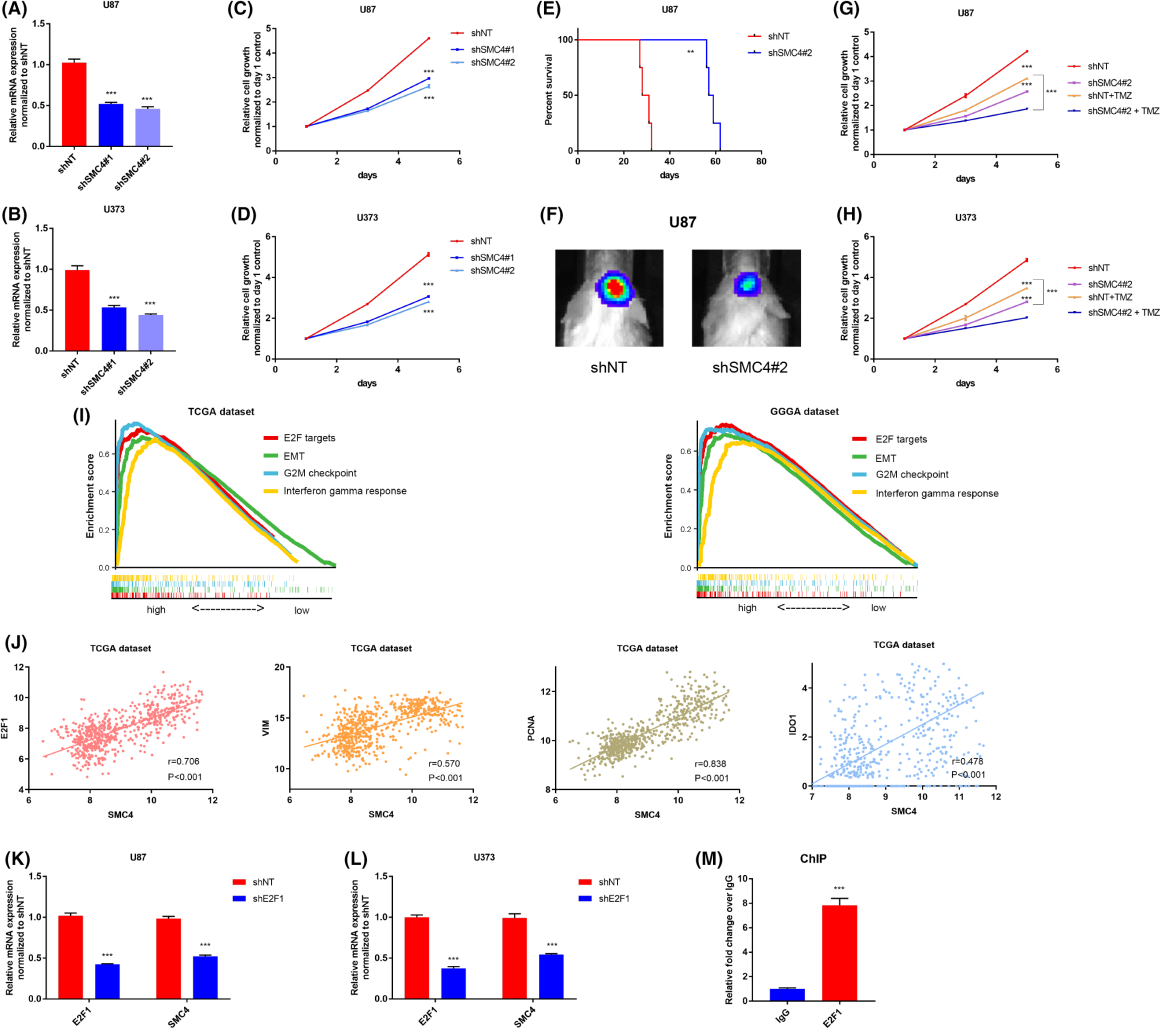
Functional verification of oncogenic effect of SMC4 in gliomas. (A, B) qRT‐PCR analysis for measuring SMC4 mRNA expression in U87 and U373 cells treated with lentiviral shSMC4#1, shSMC4#2 and shNT. (C, D) In vitro cell viability assay detecting proliferative ability of U87 and U373 cells treated with lentiviral shSMC4#1, shSMC4#2 and shNT. (E) K‐M survival analysis comparing OS for intracranial xenograft mice using U87 cells pre‐treated with shSMC4#2 and shNT. (F) Representative bioluminescence images of intracranial xenograft mice injected with luciferase‐labelled U87 cells pre‐transfected with either NT‐shRNA or SMC4‐shRNA. (G, H) In vitro cell viability assay detecting proliferative ability of U87 and U373 cells treated with/without TMZ after pre‐transfected with either shNT or shSMC4#2. (I) GSEA analyses revealing the top 4 highly enriched pathways in the high‐SMC4 expression group in TCGA and CGGA datasets. (J) Correlation analysis showed that SMC4 expression was positively related to E2F1, VIM, CDK2 and CD274 in TCGA and CGGA datasets. (K, L) qRT‐PCR analysis showed that E2F1 knockdown by lentivirus could lead to reduction of SMC4 mRNA expression in U87 and U373 cells. (M) ChIP‐PCR indicating enrichment of E2F1 at SMC4 promoter site in U87 cell. **p < 0.01, ***p < 0.001

In the DDRRG model, we found that SMC4 had the highest coefficient among the identified biomarkers. To further explore its oncogenic role in gliomas, shRNAs targeting SMC4 (shSMC4 #1 and shSMC4 #2) were introduced into U87 and U373 glioma cells. The non‐targeting (shNT) was used as control. The qRT‐PCR analysis was carried out to assess the silencing efficacy of the shSMC4 lentivirus transfection. The results indicated that SMC4 mRNA expression was significantly reduced after transfection and shSMC4 #2 had relatively higher silencing efficacy compared with shSMC4 #1 (Figure 8A,B). Next, cell viability assays were performed to explore the role of SMC4 knockdown on tumour proliferation of U87 and U373 cells. The results demonstrated that the proliferative capacity of tumour cells was significantly attenuated after SMC4 knockdown (Figure 8C,D). To comprehensively understand the aggressive roles of SMC4 on gliomas, shSMC4‐ and shNT‐bearing U87 glioma cells were intracranially injected into the brains of SCID mice. The results revealed that the SCID mice intracranially injected with shSMC4‐bearing U87 glioma cells had much better OS in comparison with those injected with shNT‐transfected cells (Figure 8E). Bioluminescent imaging further showed that SMC4 knockdown attenuated the tumorigenicity and progression of glioma cells (Figure 8F). Next, shNT‐ and shSMC4‐transfected U87 or U373 glioma cells were treated with TMZ. Cell viability assays showed that SMC4 silencing apparently improve TMZ's capability to kill glioma cells in both U87 and U373 cell lines (Figure 8G,H). In addition, to validate these results, we performed in vitro cell viability assay to detect proliferative ability of U87 tumour cells at multiple TMZ concentrations. The results suggested that silencing of SMC4 can attenuate TMZ resistance (Figure S12A). Currently, the regulation mechanism of SMC4 in gliomas is still unclear. Thus, we sought to further investigate this point. Glioma samples were classified into high‐SMC4 expression and low‐SMC4 expression groups based on median expression value of SMC4, and then, ssGSEA analysis was performed. The results revealed that E2F targets, EMT, G2M checkpoint (cell cycle) and interferon gamma response are the top enriched pathways in the high‐SMC4 expression group in both TCGA and CGGA datasets (Figure 8I). Particularly, we found BER pathway is significantly enriched in the high‐SMC4 expression group (Figure S12B). Given the result that E2F targets are one of the top activated pathways in the SMC4‐high group, we performed further expression correlation analyses for SMC4. We found that E2F1, E2F2 and E2F8 were the top three E2F family members that positively linked with SMC4. In addition, EMT, cell cycle and immune checkpoint biomarkers also demonstrated correlation with SMC4 (Figures 8J and S12C–E). Next, analyses using JASPAR dataset (http://jaspar.genereg.net/) and PROMO web tool both predicted the binding possibility for E2F1 at the potential promoter region of SMC4, but not for E2F2 and E2F8. To confirm the regulation role of E2F1 for SMC4, we performed E2F1 silencing in glioma tumour cells. qRT‐PCR results showed that E2F1 silencing significantly induces transcriptional reduction of SMC4 expression (Figure 8K–L), indicating that E2F1 is potential regulator of SMC4. To verify our hypothesis, ChIP‐PCR was performed and the results showed that E2F1 can bind to SMC4 transcription promoter region (Figure 8M). These above results suggest that SMC4 promotes glioma progression in a E2F1‐dependent manner.

## 4 DISCUSSION

Multiple studies have already demonstrated the critical links between molecular subtypes and clinical prognosis for patients with gliomas.^7^, ^8^, ^9^, ^10^ Nevertheless, molecular subtypes of gliomas have not significantly improved patients' OS.^11^ Accumulating evidence showed that disorder of DDR plays significant role in glioma progression.^39^ However, analyses focusing on DDRRGs in gliomas are still insufficient. Thus, in this study, we aimed to construct a DDRRG signature and explore the possibility of clinical application for our signature.

After comprehensive analyses, we established a risk signature that contained 16 prognosis‐related DDRRGs and provided possibility for clinical application of our risk model based on satisfactory predictive value. Among the 16 valuable biomarkers, 11 highly elevated genes in the high‐risk group, including SMC4, HEXB, VAV3, E2F7, EFNB1, WEE1, SAA1, SHISA5, PSMC2, PTGFRN and HMGA2, were significantly associated with poor outcome for patients with gliomas. Previous evidences have already confirmed the cancer‐promoting role of some biomarkers for gliomas. In addition, previous studies have reported strong links between the identified DDRRGs and TMZ resistance. For instance, a study showed that HMGA2, as a novel member of BER, directly interacts with APE1, thus causing TMZ resistance in GBM.^40^ Another study showed that SNHG12 can induce increased expression of E2F7 to promote TMZ resistance by G1/S cell cycle transition.^41^ In addition, study found that targeting G2 checkpoint kinase WEE1 can attenuate TMZ resistance in gliomas.^42^ Elevated expression of SAA1 was found to be strongly linked with TMZ resistance in a AKT dependent manner.^43^ However, more in‐depth investigations are warranted to explore the associations between DDRRGs and TMZ‐mediated therapeutic efficacy, as well as the detailed underlying mechanisms.

SMC4, which is the member of SMC gene family, plays the vital role in chromosome assembly and segregation.^44^ In addition, the complex containing SMC4, named condensin, is essential for this role.^45^ A study showed that condensin I, containing SMC4 and SMC2, is recruited to interact with base excision repair (BER) factors (PARP‐1‐XRCC1 complex), at damage sites to play in role in DNA single‐strand break (SSB) repair.^46^ Indeed, we performed GSEA analyses and found that BER activity is enriched in high‐SMC4 expression group. In addition, a study showed that TMZ can generate a series of DNA lesions, including O6‐methylguanine (Ome6G), N3‐methyladenine and N7‐methylguanine.^47^ However, N3‐methyladenine and N7‐methylguanine lesions are rapidly repair by BER pathway.^48^ Thus, higher BER activity causes resistance of temozolomide (TMZ), and targeting BER pathway is an attractive way to promote chemosensitivity.^49^, ^50^ Based on this point, we speculate that activating BER pathway is one of the critical mechanisms for SMC4 promoting TMZ resistance. Indeed, in our study, we have proved that silencing of SMC4 enhances the sensitivity of TMZ to kill U87 and U373 glioma cells. Additionally, we have proved that E2F1 is a potential transcriptional regulator for SMC4. Our study further illustrates the oncogenic role of SMC4 for GBM on the basis of previous studies.^51^

Another noteworthy finding was that there was distinct enrichment of immune‐related pathways between high‐risk and low‐risk groups. In addition, we used CIBERSORT and ssGSEA to explore immune infiltrations, and the results showed that the high‐risk group is positively related to immunosuppressive cell types, such as M2‐type macrophages,^33^ Tregs and MDSC.^34^ For further verification, we carried out comparison and correlation analyses. The results showed that secreted immune inhibitory factors and immune checkpoint markers have elevated expression in the high‐risk group. Previous studies have well documented the immunosuppressive effects of these biomarkers. For instance, the study showed that PD‐L1 derived from tumour could inhibit T cell responses though binding with PD‐1 expressed by T cells.^52^ Interestingly, recent evidence showed that PD1^+^TAM population expressed an M2‐like surface profile to inhibits phagocytosis and immune response.^53^ Besides, a study showed that Tregs could express CTLA‐4 to elicit suppression^54^; meanwhile, CTLA‐4 itself could play an inhibitory role by triggering inhibitory signals.^55^ In accordance with previous findings, we found that high‐risk score is negatively associated with activated CD8^+^ T cells. These results show that high‐risk group is strongly linked with immunosuppressive microenvironment caused by interaction of multiple immunosuppressive factors. Based on this point, we analysed the differences of therapeutic response between high‐risk and low‐risk groups. The results showed that high‐risk score is resistant to ICB therapy. Accumulating evidences have illustrated that higher mutational load is linked with satisfactory objective response to immunotherapy in non‐small cell lung cancer (NSCLS)^36^ and metastatic melanoma.^35^ However, recent study showed that clinical response to anti‐PD‐1 immunotherapy in GBM is linked with lower TMB.^32^ In addition, recent evidence found that low mutation burden is linked with response to immunotherapy in recurrent GBM.^56^ Moreover, a study reported that PTEN mutation is associated with immunotherapy resistance in gliomas.^32^, ^57^ In our study, we found that the high‐risk group had higher TMB and more frequent PTEN mutation. Meanwhile, we observed that high‐risk score is a resistant phenotype for immunotherapy in gliomas via TIDE database and GenePattern database. This consistency between our results and previous findings further confirms the value of applying our model in the clinical.

We analysed the DDRRG signature from multiple aspects and observed more satisfactory value in comparison with previous glioma signature.^58^, ^59^, ^60^ Nevertheless, we must point out certain shortcomings and limitations in our study. Firstly, the main source of this study was downloaded from available public databases. Although we preliminary verified the oncogenic role of SMC4 in vitro and in vivo, multiple key factors should be further validated. For instance, the highly expressed immune infiltrations such as Tregs and M2 macrophages need to be further studied. Secondly, although we preliminarily analysed potential response to ICB in gliomas, the reference expression profiles are from melanoma, so we still lack cohorts for clinical sample from glioma patients undergoing immunotherapy. Thirdly, detailed clinical data are warranted to more accurately assess the therapeutic response of glioma sample, such as information of treatment dose and time after standard surgical resection treatment.

In conclusion, we aimed to understand DDRRGs regulation in gliomas. Ultimately, we identified and validated a 16‐gene signature from multiple aspects by comprehensive analyses. This risk model facilitates robust predictive ability and harbours robust risk stratification ability for OS of glioma patients. In addition, this risk model is strongly linked with multiple oncogenic pathways, immunosuppressive tumour microenvironment and therapeutic response.

## AUTHOR CONTRIBUTIONS

**Xiaodong Li:** Conceptualization (equal); data curation (lead); formal analysis (lead); software (lead); visualization (lead); writing – original draft (lead). **Yichang Wang:** Data curation (supporting); formal analysis (supporting); methodology (supporting); software (supporting); validation (supporting). **Wei Wu:** Data curation (supporting); methodology (equal); software (supporting); validation (supporting); visualization (supporting). **Jianyang Xiang:** Methodology (supporting); validation (equal); visualization (supporting). **Maode Wang:** Conceptualization (lead); project administration (lead); resources (lead); supervision (lead). **Hai Yu:** Conceptualization (lead); formal analysis (lead); investigation (lead); project administration (lead); software (equal); supervision (lead); validation (lead).

## CONFLICT OF INTEREST

The authors confirm that there are no conflicts of interest.

## Supporting information


Table S1

Table S2

Table S3

Table S4

Figure S1‐S12

Data S1

## Data Availability

All data used in this study can be downloaded from TCGA dataset (https://xenabrowser.net/datapages/) and the CGGA dataset (http://www.cgga.org.cn).
