# Supplementary material for: A novel DNA damage and repair‐related gene signature to improve predictive capacity of overall survival for patients with gliomas
Source: J Cell Mol Med. 2022 May 26;26(13):3736–50. doi: 10.1111/jcmm.17406 (PMC9258707; doi:10.1111/jcmm.17406)
Supplement: Supplementary file 1 — Table S1 [file JCMM-26-3736-s003.docx]

**Table S1. Characteristics of patients in cluster 1 and cluster 2 in the CGGA dataset**

| **Characteristics** | **N** | **Cluster1** | **Cluster2** | **p value** |
| --- | --- | --- | --- | --- |
| **Total cases** | 309 | 182 | 127 |  |
| **Gender** |  |  |  | 0.170 |
| Male | 194 | 120 | 74 |  |
| Female | 115 | 62 | 53 |  |
| **Age (years)** |  |  |  | <0.001 |
| ≤42 | 155 | 72 | 83 |  |
| ＞42 | 154 | 110 | 44 |  |
| **Grade** |  |  |  | <0.001 |
| II | 97 | 13 | 84 |  |
| III | 73 | 44 | 29 |  |
| IV | 135 | 121 | 14 |  |
| **IDH Status** |  |  |  | <0.001 |
| Mutation | 165 | 54 | 111 |  |
| Wildtype | 143 | 128 | 15 |  |
| **MGMT Promoter** |  |  |  | 0.036 |
| Methylation | 151 | 81 | 70 |  |
| Unmethylation | 140 | 92 | 48 |  |
| **1p19q** |  |  |  | <0.001 |
| Codel | 62 | 2 | 60 |  |
| Non-codel | 239 | 174 | 65 |  |
