# Supplementary material for: A novel DNA damage and repair‐related gene signature to improve predictive capacity of overall survival for patients with gliomas
Source: J Cell Mol Med. 2022 May 26;26(13):3736–50. doi: 10.1111/jcmm.17406 (PMC9258707; doi:10.1111/jcmm.17406)
Supplement: Supplementary file 2 — Table S2 [file JCMM-26-3736-s005.docx]

**Table S2.** **LASSO regression coefficients of 16 survival-related DDRRGs in gliomas**

| **Gene** | **value** |
| --- | --- |
| FBXO18 | -0.0579 |
| MMS19 | -0.0029 |
| SMC4 | 0.2184 |
| HEXB | 0.0478 |
| UBQLN4 | -0.0036 |
| VAV3 | 0.0066 |
| E2F7 | 0.0149 |
| EFNB1 | 0.0134 |
| WEE1 | 0.0875 |
| SAA1 | 0.0391 |
| SHISA5 | 0.1342 |
| WAC | -0.0122 |
| PSMC2 | 0.1369 |
| PTGFRN | 0.0707 |
| EIF3L | -0.0492 |
| HMGA2 | 0.0272 |
