# Supplementary material for: A novel DNA damage and repair‐related gene signature to improve predictive capacity of overall survival for patients with gliomas
Source: J Cell Mol Med. 2022 May 26;26(13):3736–50. doi: 10.1111/jcmm.17406 (PMC9258707; doi:10.1111/jcmm.17406)
Supplement: Supplementary file 4 — Table S4 [file JCMM-26-3736-s004.docx]

**Table S4. Univariate and multivariate analyses of risk score and clinical characteristics in the CGGA dataset**

| **Variables** | | **Univariate analysis** | | **Multivariate analysis** | |
| --- | --- | --- | --- | --- | --- |
|  |  | **HR (95% CI)** | **p value** | **HR (95% CI)** | **p value** |
| **Risk score** | 4.060(3.187-5.172) | | <0.001 | 2.789(1.869-4.102) | <0.001 |
| **Age** | 1.033(1.019-1.046) | | <0.001 | 1.012(1.000-1.025) | 0.059 |
| **Gender** | 0.995(0.748-1.323) | | 0.973 |  |  |
| **WHO Grade** |  | |  |  |  |
| **III** | 3.138(2.010-4.900) | | <0.001 | 2.339(1.470-3.724) | <0.001 |
| **IV** | 8.096(5.377-12.191) | | <0.001 | 3.497(2.157-5.670) | <0.001 |
| **IDH mutation** | 0.305(0.261-0.469) | | <0.001 | 1.898(1.253-2.876) | 0.003 |
| **MGMT status** | 0.885(0.669-1.169) | | 0.389 |  |  |
| **1p19q status** | 0.183(0.112-0.299) | | <0.001 | 0.315(0.184-0.538) | <0.001 |
