# Supplementary material for: A novel DNA damage and repair‐related gene signature to improve predictive capacity of overall survival for patients with gliomas
Source: J Cell Mol Med. 2022 May 26;26(13):3736–50. doi: 10.1111/jcmm.17406 (PMC9258707; doi:10.1111/jcmm.17406)
Supplement: Supplementary file 5 — Figure S1‐S12 [file JCMM-26-3736-s002.pdf]

Supplementary figures

Figure S1

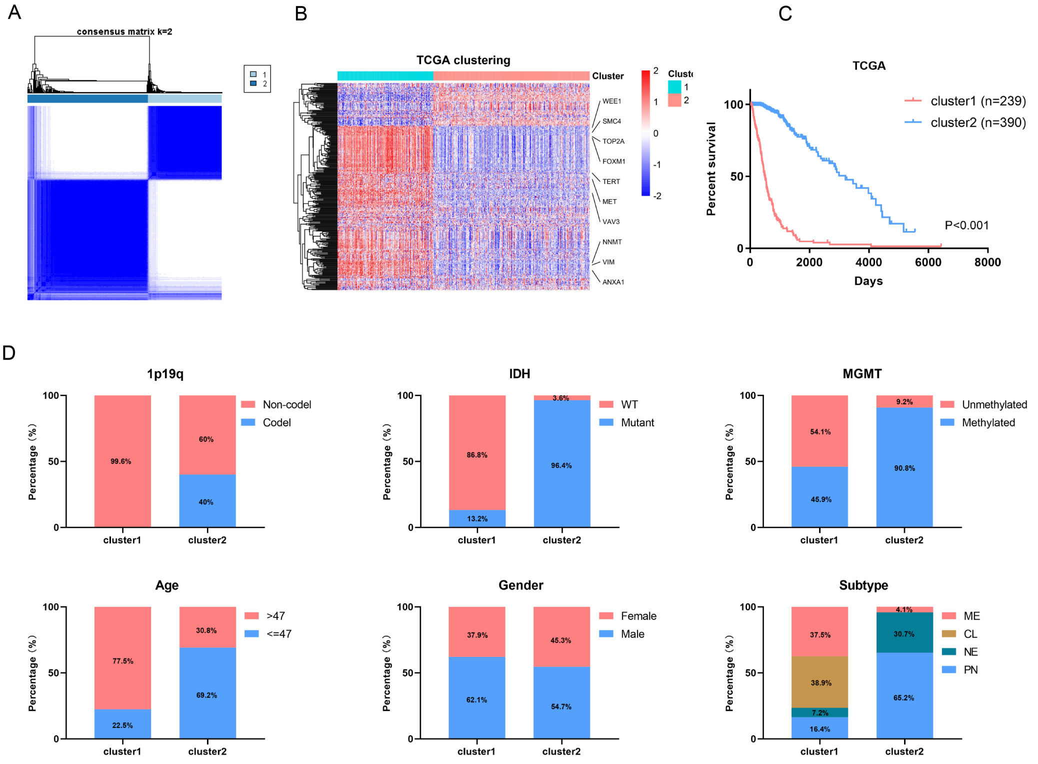

Figure S1. Using the DDRRGs for the consensus clustering of gliomas in TCGA dataset

(**A**) 629 samples from TCGA dataset were divided into 2 distinct clusters. (**B**) The heatmap of 2 clusters showing distinct expression patterns of the top 300 differential DDRRGs. (**C**) The K-M curve showed that the OS of cluster 1 was apparently poorer than that of cluster 2. (**D**) Percent stacked column charts showing proportion of patients with distinct clinical feature for cluster 1 and cluster 2 in TCGA dataset.

Figure S2

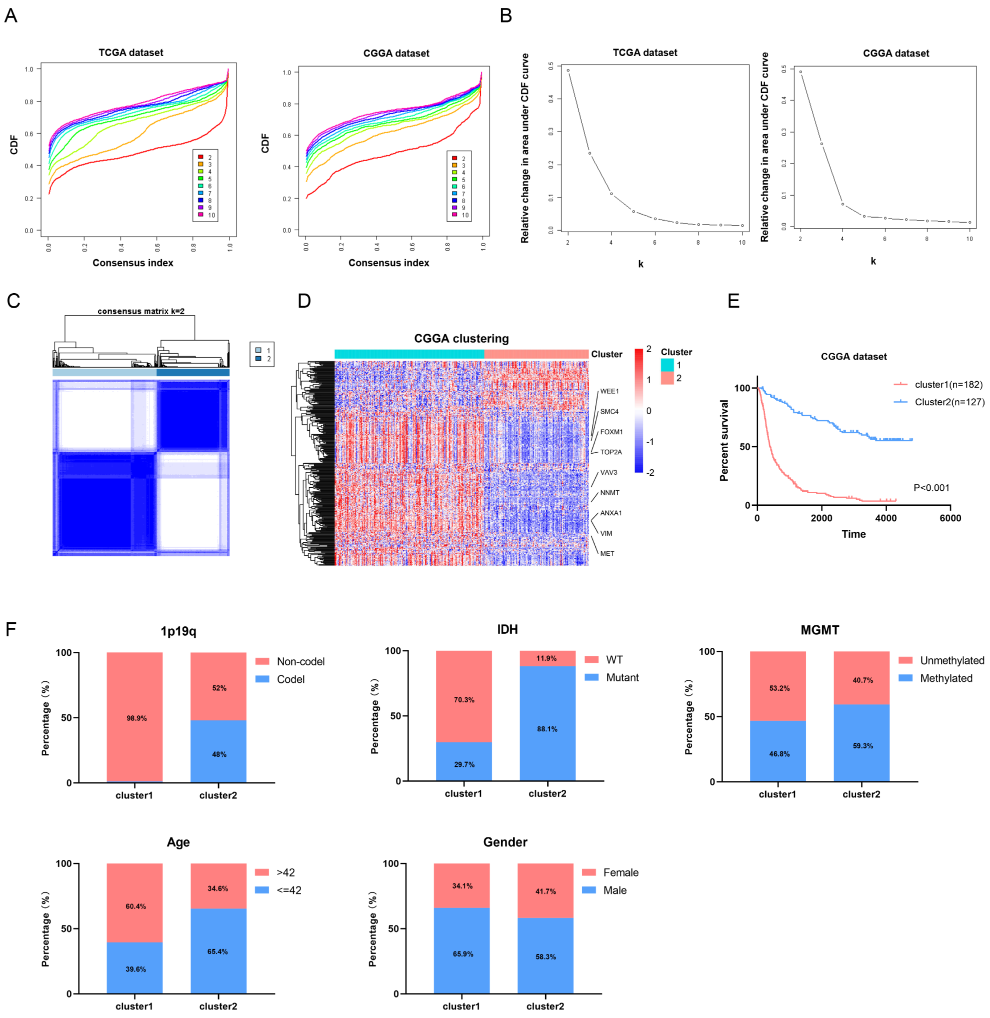

Figure S2. Using the DDRRGs for the consensus clustering of gliomas in the CGGA dataset

(**A**) CDF curve for k = 2 to k = 10 in TCGA and CGGA datasets. (**B**) Relative change under CDF delta area curve for k = 2 to k = 10 in TCGA and CGGA datasets. (**C**) 309 samples from the CGGA dataset were divided into 2 distinct clusters. (**D**) The heatmap of 2 clusters showing distinct expression patterns of the top 300 differential DDRRGs. (**E**) The K-M curve showed that the OS of cluster 1 was apparently poorer than that of cluster 2. (**F**) Percent stacked column charts showing proportion of patients with distinct clinical feature for cluster 1 and cluster 2 in the CGGA dataset.

Figure S3

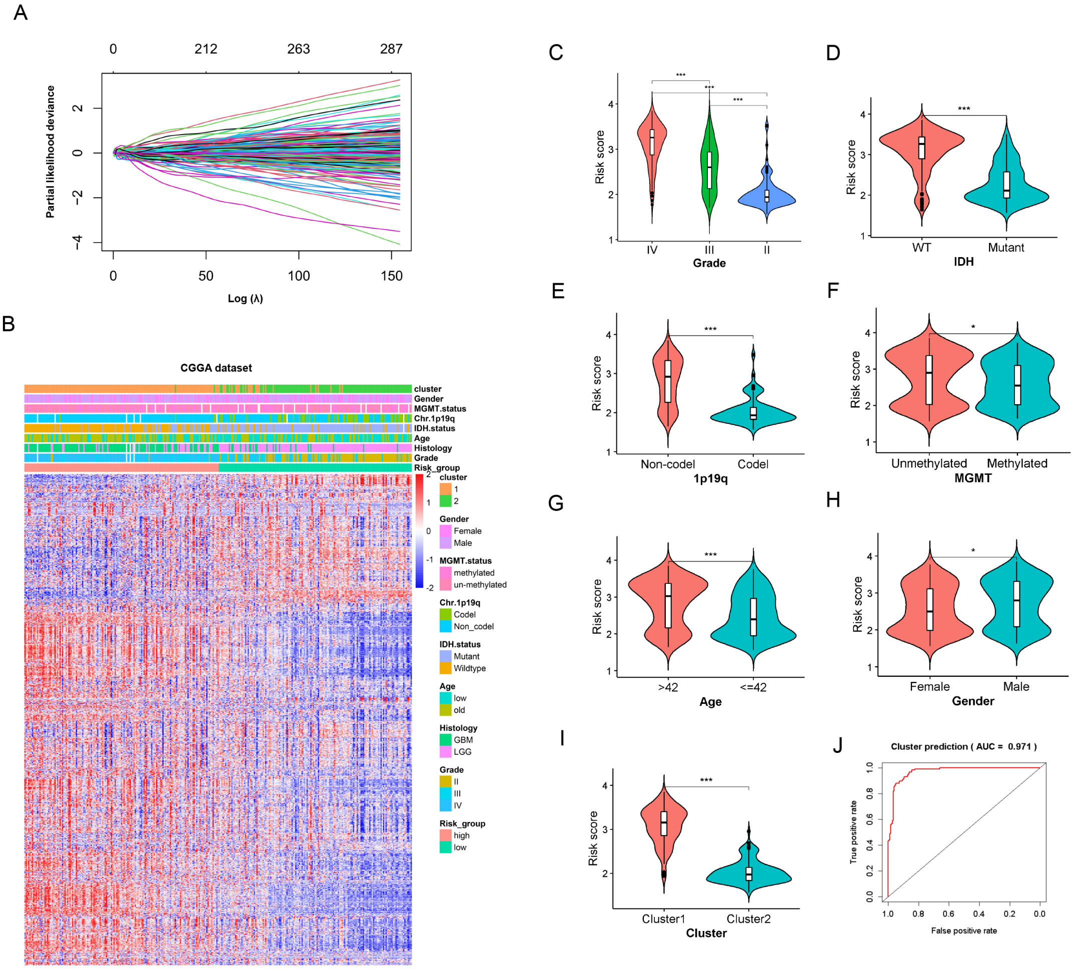

Figure S3. Exploration of the clinical characteristics of the DDRRG signature in gliomas in the CGGA dataset

(**A**) LASSO coefficient profiles of the most robust prognostic-related genes. (**B**) The heatmap showing the expression features of 1547 DDRRGs and corresponding clinical patterns. (**C-I**) Violin plots comparing the risk score for patients with gliomas sub-grouped by WHO grade, IDH mutation status, 1p/19q status, MGMT promoter methylation status, age, gender or cluster of gliomas. (**J**) ROC curve showing the predictive ability of the DDRRG signature for the clusters. * P < 0.05, *** P < 0.001.

Figure S4

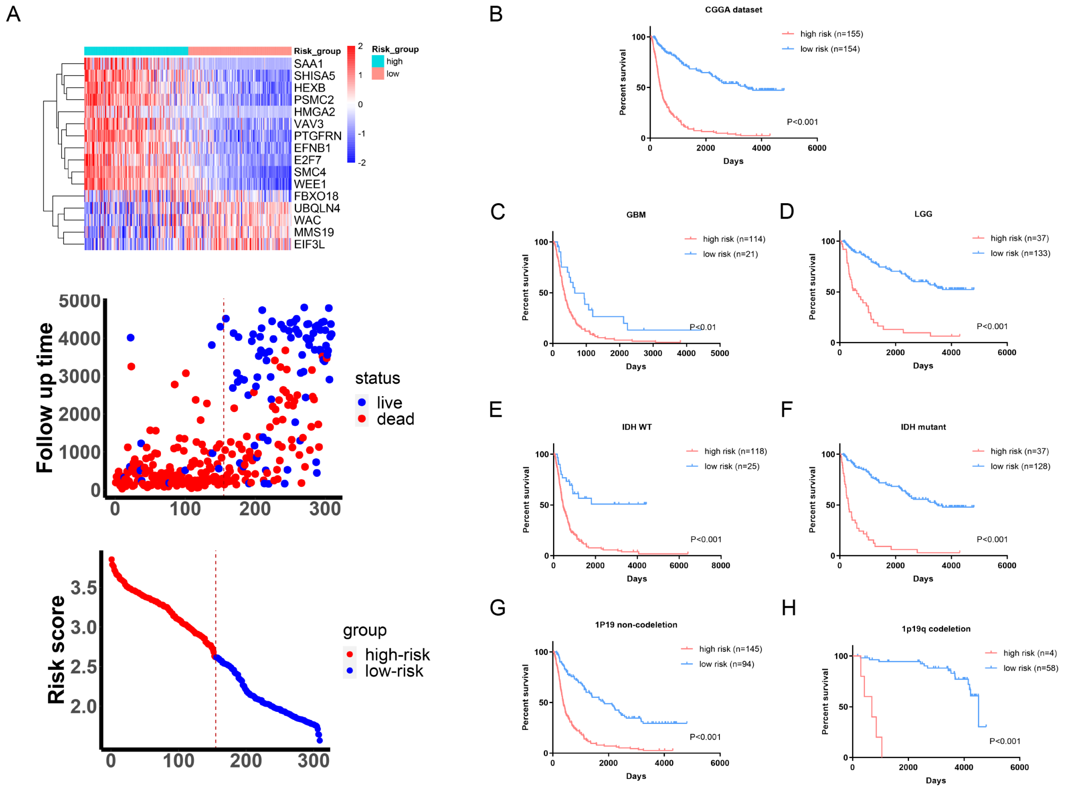

Figure S4. The prognostic value of the DDRRG model in the CGGA dataset

(**A**) Distribution of the risk score, expression level of 16 genes and prognostic status between high-risk and low-risk groups. (**B-H**) K-M curves comparing prognosis for patients with gliomas sub-grouped by grade, IDH mutation or 1p/19q status between high-risk and low-risk groups.

Figure S5

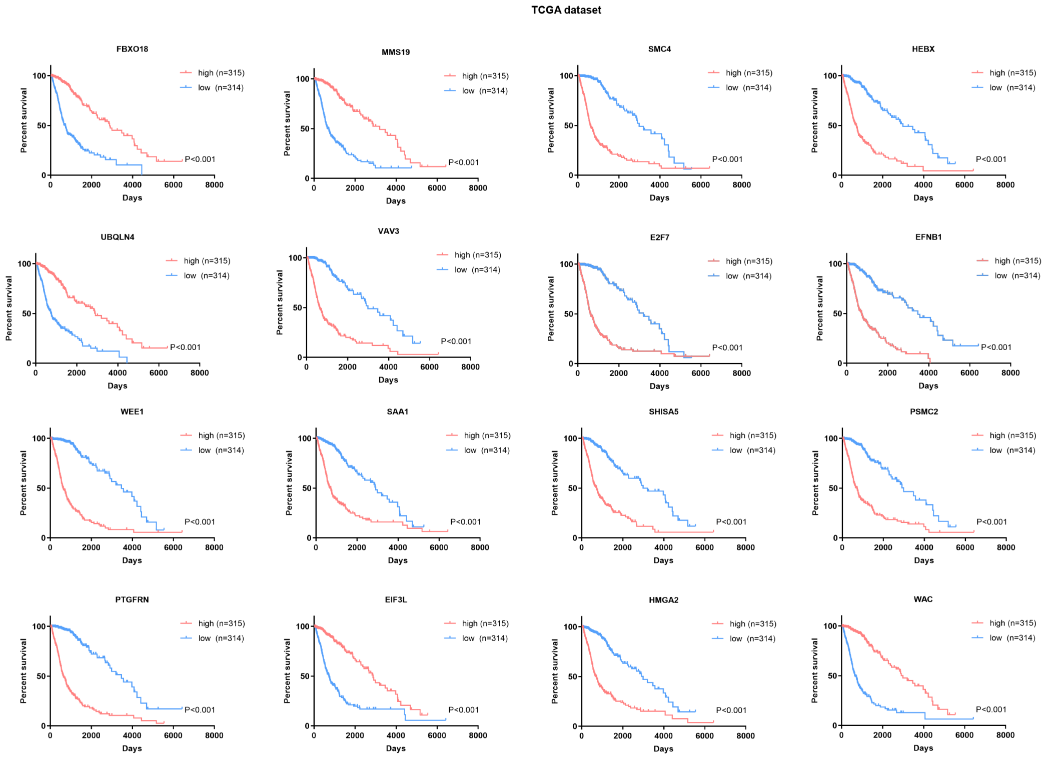

Figure S5. The prognostic value of 16 DDRRGs in TCGA dataset

K-M curves showing outcome of glioma patients based on the median value of 16 gene expression between high-expression and low-expression groups in TCGA dataset.

Figure S6

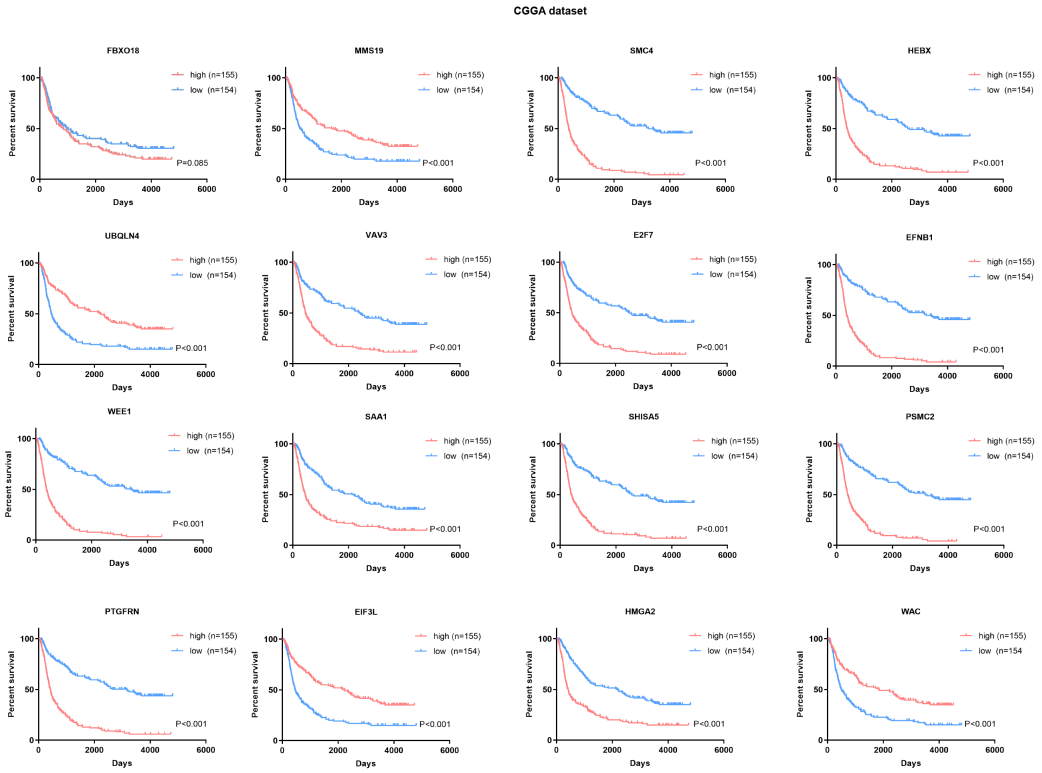

Figure S6. The prognostic value of 16 DDRRGs in the CGGA dataset

K-M curves showing outcome of glioma patients based on the median value of 16 gene expression between high-expression and low-expression groups in the CGGA dataset.

Figure S7

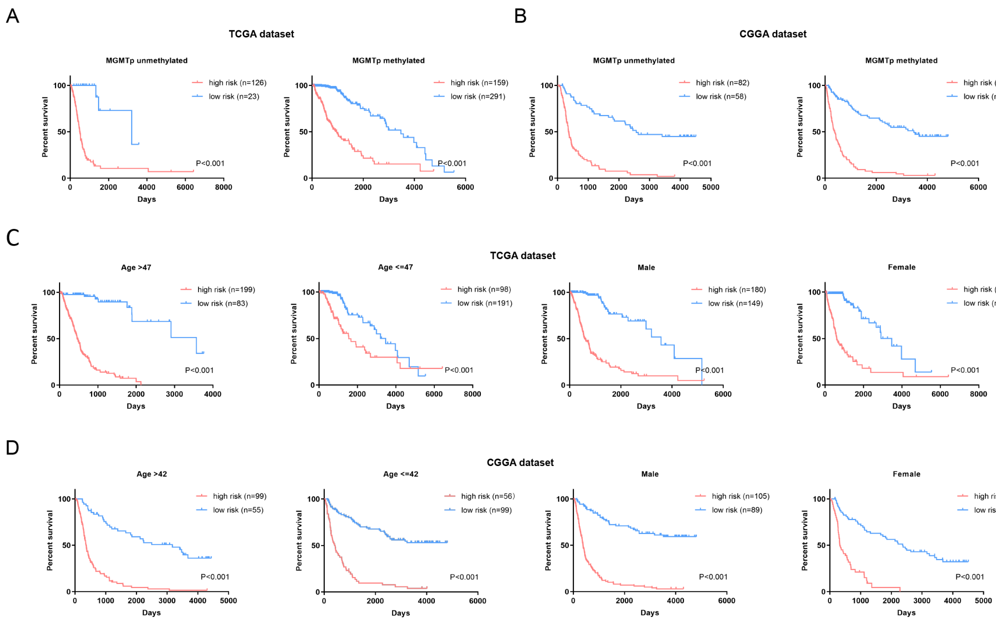

Figure S7. The prognostic value of the DDRRG signature for the patients sub-grouped by MGMT promotor methylation status, age or gender

K-M curves showing outcome for glioma patients between high-risk and low-risk groups sub-grouped by MGMT promotor methylation status, age or gender in TCGA (**A, C**) and CGGA datasets (**B, D**), respectively.

Figure S8

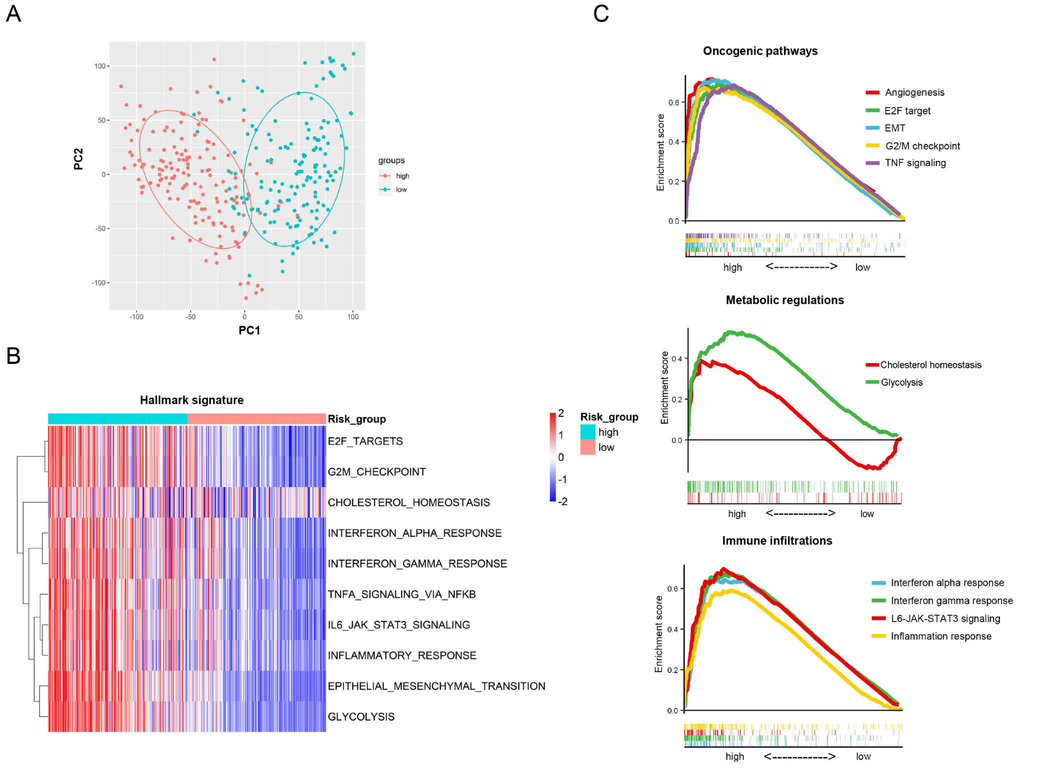

Figure S8. Functional analyses of the DDRRG signature in the CGGA dataset

(**A**) PCA analysis showing distinct gene expression distribution between high- and low-risk groups. (**B**) GSVA analysis was performed to evaluate meta-score of hallmark signature in each sample of gliomas in the CGGA dataset. The heatmap showing meta-score of each hallmark signature between high-risk and low-risk groups. (**C**) GSEA analyses revealing the highly enriched hallmark signatures, metabolic pathways and immune infiltrations in the high-risk group.

Figure S9

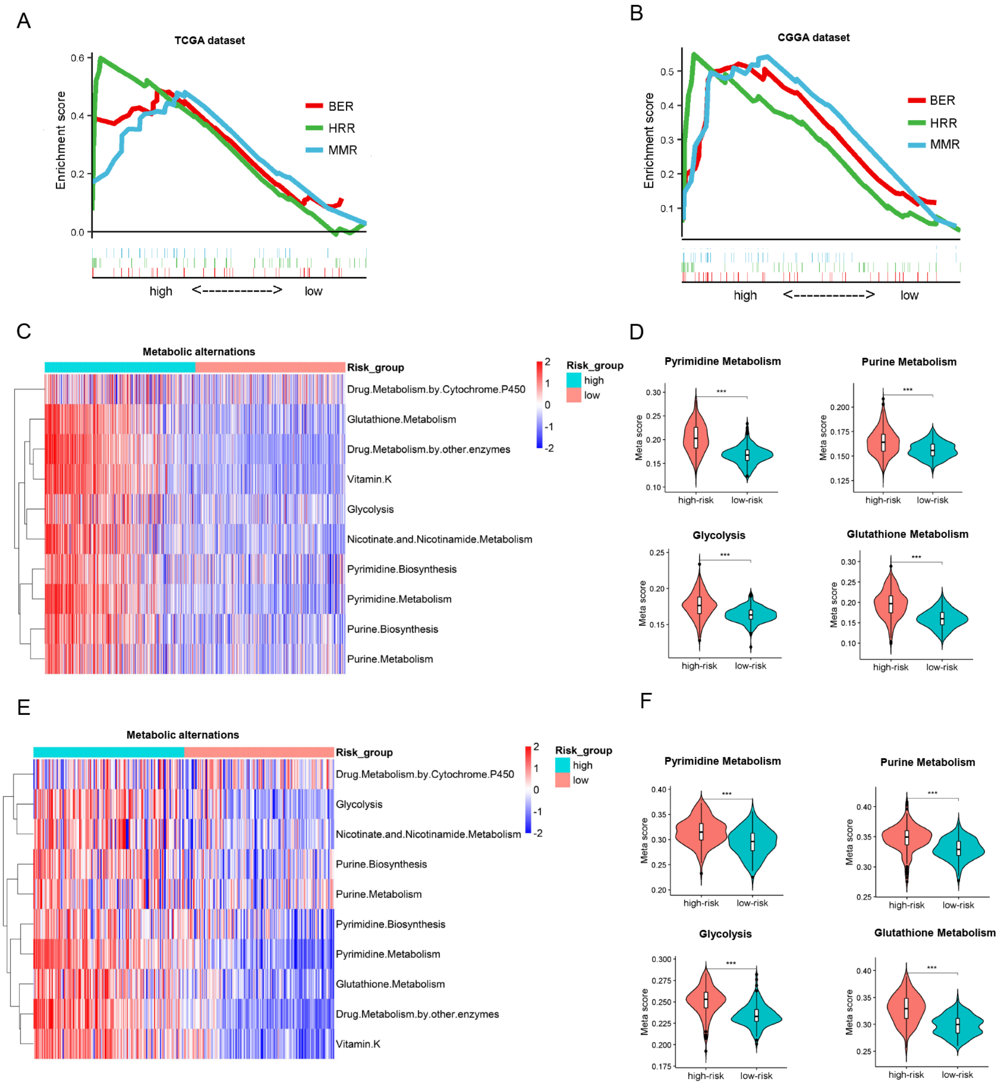

Figure S9. Exploration of differences of DNA repair type and metabolic alternations between high-risk and low-risk group

(**A-B**) GSEA analyses revealing the highly enriched DNA repair types in the high-risk group in TCGA and CGGA datasets. (**C, E**) The heatmap showing 10 glioma-related metabolic pathways that are enriched in the high-risk group in TCGA (**C**) and CGGA (**E**) datasets. (**D, F**) Violin plots comparing the meta-score of glioma-related metabolic alternations between high- and low-risk groups in TCGA (**D**) and CGGA (**F**) datasets. *** $P < 0.001$.

Figure S10

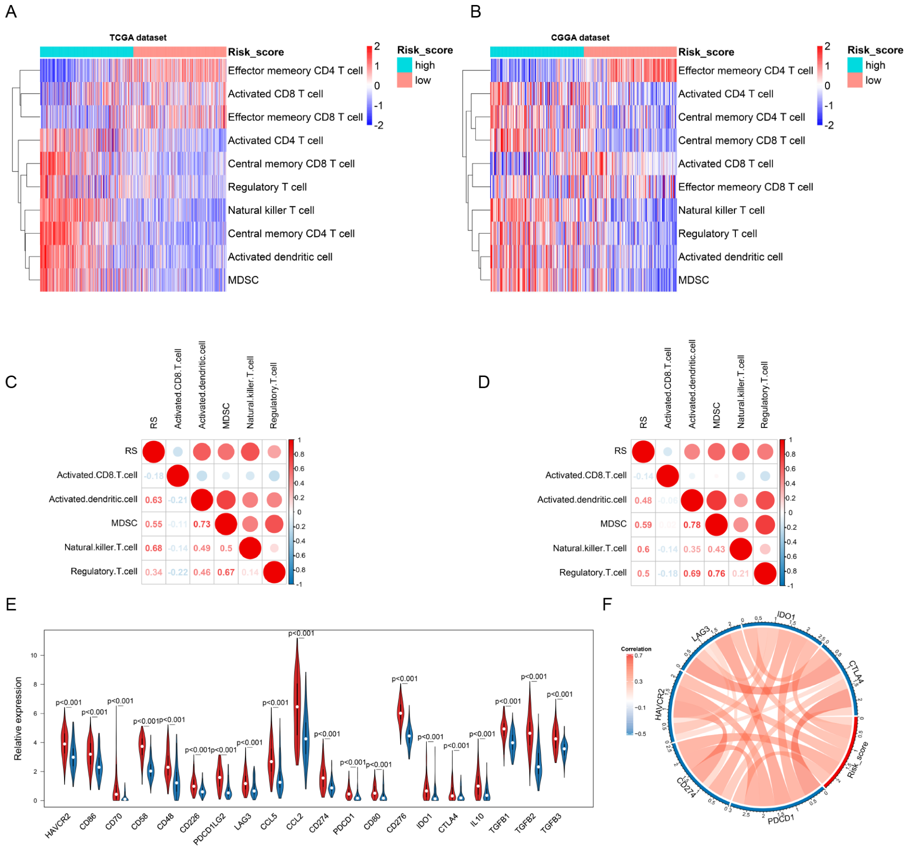

Figure S10. High-risk score is closely linked with immunosuppressive microenvironment of gliomas

(**A, B**) ssGSEA analysis was carried out to evaluate the infiltration of 28 immune cell population in TCGA and CGGA datasets. The heatmap showing ssGSEA score for each immune cell population. (**C, D**) The correlation analysis showing the association between the immune infiltration level and the risk score in TCGA and CGGA datasets. (**E**) Comparison analyses showing expression level of immunosuppressive checkpoints between high-risk and low-risk groups in the CGGA dataset. (**F**) CIRCOS plot showing the relationship between immune checkpoint markers and the risk score in the CGGA dataset.

Figure S11

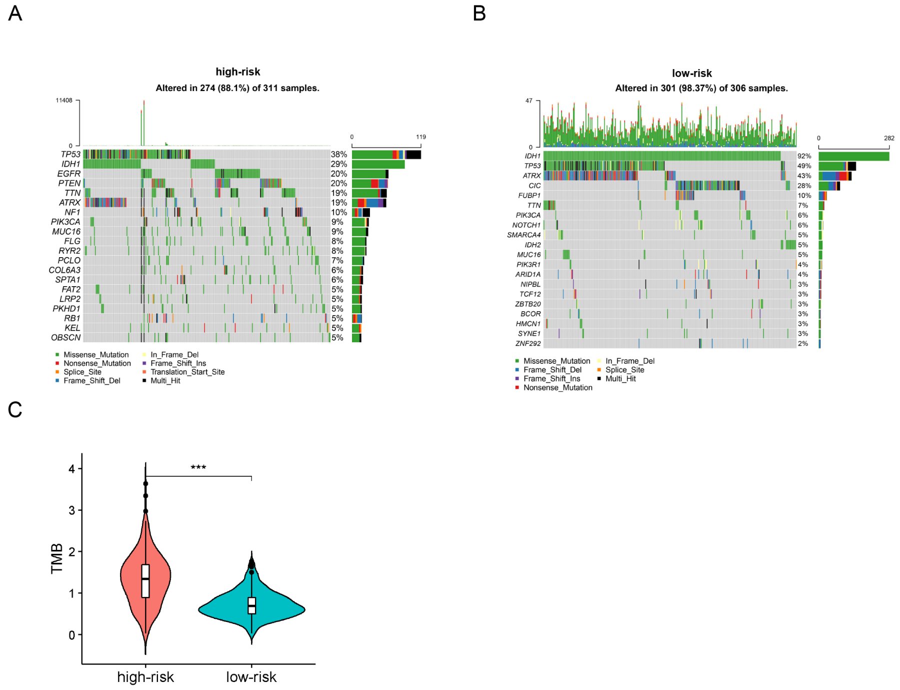

Figure S11. Distinct somatic mutation burden landscape between high-risk and low-risk groups

(**A, B**) Waterfall of the top 20 mutated genes between high-risk and low-risk groups.

(**C**) Violin plot comparing TMB between high-risk and low-risk groups. *** P < 0.001.

Figure S12

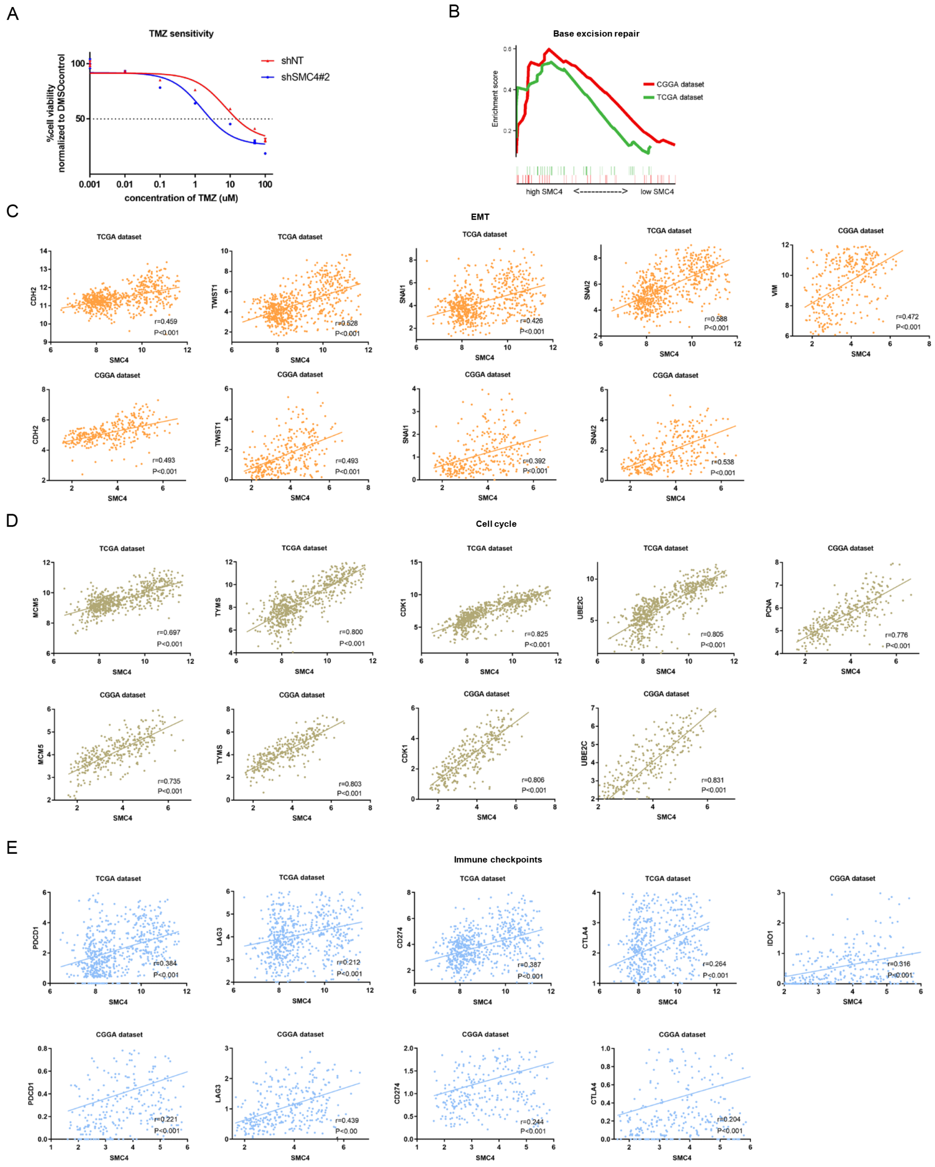

Figure S12. Functional verification of oncogenic role of SMC4 in gliomas

(**A**) *In vitro* cell viability assay detecting the proliferative ability of U87 glioma cells pre-transfected with either shNT or shSMC4#2 at multiple TMZ concentrations. (**B**) GSEA analyses revealing the enrichment results of base excision repair in the high-SMC4 and low-SMC4 expression groups in TCGA and CGGA datasets. (**C-E**) The correlation analysis between SMC4 and representative factors of EMT, cell cycle and immune checkpoint genes in TCGA and CGGA datasets.
