# Supplementary material for: A novel DNA damage and repair‐related gene signature to improve predictive capacity of overall survival for patients with gliomas
Source: J Cell Mol Med. 2022 May 26;26(13):3736–50. doi: 10.1111/jcmm.17406 (PMC9258707; doi:10.1111/jcmm.17406)
Supplement: Supplementary file 6 — Data S1 [file JCMM-26-3736-s001.docx]

**Supplementary materials and methods**

**Included patients and datasets**

Level 3 mRNA expression profiles integrated by the Illumina HiSeq RNASeqV2 system from 629 samples in TCGA dataset were obtained from TCGA project (<https://xenabrowser.net/datapages/>). The detailed data of 309 samples were obtained from the CGGA dataset (<http://www.cgga.org.cn>). The normalized count reads from the pre-processed data, including sequence alignment and transcript abundance estimation, were log2 transformed. The clean reads were aligned to human genome reference (hg19) and RSEM was used to calculate sequencing read counts for each RefSeq gene. TCGA dataset was included as the discovery cohort, and the CGGA dataset was used as validation cohort.

**Consensus clustering**

Median absolute deviation (MAD) was used to identify the most variable genes that was further used for consensus clustering. Using R package “ConsensusClusterPlus” (1.54.0), we carried out consensus clustering analysis.

**Nomogram construction**

R package “rms” (6.2.0) was used to construct the predictive model incorporating the risk score and clinicopathologic parameters (age and 1p19q codeletion status). Calibration curve was applied to assess the consistence of the nomogram. Harrell's concordance index (Harrell’s C-index) was calculated to evaluate the discrimination of the nomogram.

**Pathway activation analyses**

Principal component analysis (PCA): R package “princomp” was used to identify the distinct distribution of DDRRGs between high-risk and low-risk groups.

Gene set variation analysis (GSVA) is a nonparametric and unsupervised gene set enrichment method that could estimate the enrichment score (meta-score) of certain signature or pathway based on transcriptional expression data of samples^[1]^. Firstly, we obtained 50 hallmark gene profiles (<http://www.gsea-msigdb.org/>) and 114 metabolic pathways from previously studies^[2]^. In total, 164 scores for each sample corresponding to 50 hallmark signatures and 114 metabolic pathways by using “GSVA” package (1.38.2). R package “pheatmap” (1.0.12) was used to visualize the results.

Gene set enrichment analysis (GSEA) was performed through a well-known online network (<http://software.broadinstitute.org/gsea/index.jsp>). GSEA software (4.1.0) used normalized values for gene expression as input file. P values were calculated after performing 1,000 permutations. The h.all.v7.4.symbols.gmt [Hallmarks] from Molecular Signatures Database (MSigDB) was used as the reference gene set. Gene sets with a false discovery rate (FDR) value <0.05 were considered significant enrichment^[3]^.

**Analyses of immune signature**

CIBERSORT, a computational deconvolution method, was used to estimate the absolute fractions of tumor infiltrating immune cells (TIICs) in the gene expression profiles (GEPs) of a mixed cell population^[4]^. LM22, containing 547 genes that could identify 22 human hematopoietic cell populations, was used as reference file (https://cibersortx.stanford.edu/). R package “CIBERSORT” was used to calculate the proportions of 22 TIICs for each sample by performing 1,000 permutations. Simultaneously, pearson correlation coefficient and root mean squared error (RMSE) were computed for each sample. Significant samples (*P* < 0.05) were selected for subsequent analysis. The results were visualized via “pheatmap” package (1.0.12).

Additionally, the 782 metagenes for determining 28 immune cell subpopulations were download from another previous study^[5]^. Single-sample gene set enrichment analysis (ssGSEA) was performed via R packages “GSVA” (1.38.2) to calculate meta-score and quantify the immune infiltration levels. Unsupervised clustering was carried out by the meta-scores obtained from TCGA and CGGA datasets and visualized via R packages “pheatmap” (1.0.12) and “corrplot” (0.84)^[6]^.

**Analyses of immunotherapy response**

The Tumor Immune Dysfunction and Exclusion (TIDE) is a computational framework to assess the potential mechanisms of tumor immune evasion^[7]^. We used online TIDE database (<http://tide.dfci.harvard.edu/>) to predict the response to immune checkpoint blockade (ICB) in gliomas. In addition, we used subclass mapping algorithm to further validate the response to ICB^[8]^. The expression data of GSE78220^[9]^ , containing 28 patients with metastatic melanoma that underwent PD-1 checkpoint inhibition therapy, was derived from Gene Expression Omnibus (GEO) dataset and was used as reference file. Informed consents from patients were obtained in this study. The normalized values for gene expression of gliomas from TCGA and CGGA datasets were used as input files for above two methods.

**Estimation of tumor mutational burden (TMB)**

TMB was defined as the number of non-synonymous somatic gene alterations per megabase (Mb) in the coding region, and was computed as (whole counts of exome non-synonymous gene variants)/ (38 Mb)^[10]^. The mutational data of gliomas was downloaded from TCGA project (<https://portal.gdc.cancer.gov/>). R package "maftools" (2.6.05) was applied to analyze mutation data and generate graphics. TMB of each sample was automatically computed via Perl script (<https://www.perl.org/>). Patients was assigned into the high- and low-TMB groups based on the median cutoff value of TMB.

**Reagents and Antibodies**

Following reagents were used: DMEM-F12, Fetal bovine serum (FBS), Penicillin-Streptomycin, Trypsin-EDTA (Thermo scientific), anti-rabbit IgG-Horseradish peroxidase (NA934V), anti-mouse IgG-Horseradish peroxidase (NXA931) (GE Healthcare).

***In vitro* cell cultures**

U87 and U373 glioma cell lines were provided by the First Affiliated Hospital of Xi'an Jiaotong University. Tumor cells were cultivated in DMEM-F12 medium containing FBS supplement (10% vol) and Penicillin-Streptomycin antibiotics (1%). The culture medium was changed every 3-4 days.

**RNA Isolation and Quantitative Real-Time Polymerase Chain Reaction (qRT-PCR)**

The qRT-PCR analysis was performed based on methods as previously described ^[11]^. The primer sequences applied in the study include the following:

SMC4 (forward CGCCTCCAGCAATGACCAAT; reverse CCCCAGCATAGGATTTGAAGTT)

E2F1 (forward ACGCTATGAGACCTCACTGAA; reverse TCCTGGGTCAACCCCTCAAG)

GAPDH (forward: GGAGCGAGATCCCTCCAAAAT; reverse: GGCTGTTGTCATACTTCTCATGG)

**Cell** **viability assay**

Viability of glioma cells was detected using AlamarBlue reagent (Thermo scientific). Glioma cells were seeded into a 96 well plate at the density of 1*10^3^ cells per well and cultured in the medium described above. Then each well was added with AlamarBlue reagent and fluorescence was measured (Excitation 515-565 nm, Emission 570-610 nm) after 6 hours using Synergy HTX multi-mode reader (BioTek).

***In vivo* intracranial xenograft tumor models**

The xenograft model was constructed using 6-8 weeks old severe combined immune-deficiency (SCID) mice through the method as previously described^[11]^. For *in vivo* bioluminescent imaging, XenoLight D-luciferin (PerkinElmer) solution (2.5 mg/100ul) was injected into animals intraperitoneally, and then mice were anesthetized with isoflurane for the imaging analysis using an IVIS 100 imaging system (PerkinElmer) to capture the luciferase images.

**Lentivirus production and transduction**

Lentivirus infection was performed using the method as previously described^[11]^. Target sequence for used shRNA in this study:

shSMC4#1: GCCACAAGAGTAGCATATCAA;

SHSMC4#2: GCCCAACAAGACAAACTTGAT.

**Chromatin immunoprecipitation**

Chromatin immunoprecipitation (ChIP) was carried out according to the manufacturer's protocol. Bioruptor UCD-200 was applied for sonication of DNA, and 2*10^6^ cells were used for performing following each reaction. Promoter sequence (SMC4): forward: CAGGTCTCCGGAATGCGTAA, reverse: GGGAAGTCGCCACACATTCT.

**Statistics**

R packages used in this study: “pheatmap” package (1.0.12) for visualization of expression heatmap, “glmnet” package (4.1-1) for Lasso analysis, "ggpubr" package (0.4.0) for violin plot visualization, “survivalROC” (1.0.3) and “pROC” package (1.17.0.1) for ROC analysis, “GSVA” package (1.38.2) for analysis hallmark signature, metabolic alternations and immune infiltrations, “corrplot” package (0.84) for visualization of correlation heatmap, “circlize” package (0.4.12) for circle plot visualization. Chi-square test was applied to identify the distinct distribution of clinicopathologic features between different clusters. Univariate and multivariate Cox regression analyses were performed to assess the independent prognostic value for each variable. Two tailed t-test was carried out to assess statistical significance within two groups. The Kaplan-Meier (K-M) analysis was carried out to compare OS of patients in different groups. Patients were assigned into two groups based on the median value of each gene expression or risk score for prognostic analysis. Correlation analysis was used to calculate Pearson's correlation coefficient. This study was approved by the Institutional Review Boards (IRB) of the First Affiliated Hospital of Xi’an Jiaotong University (XJTU).

**Reference**

1. Hänzelmann S, Castelo R, Guinney J. GSVA: gene set variation analysis for microarray and RNA-seq data. BMC Bioinformatics. 2013;14:7.

2. Rosario SR, Long MD, Affronti HC, Rowsam AM, Eng KH, Smiraglia DJ. Pan-cancer analysis of transcriptional metabolic dysregulation using The Cancer Genome Atlas. Nat Commun. 2018;9(1):5330.

3. Subramanian A, Tamayo P, Mootha VK, Mukherjee S, Ebert BL, Gillette MA, et al. Gene set enrichment analysis: a knowledge-based approach for interpreting genome-wide expression profiles. Proc Natl Acad Sci U S A. 2005;102(43):15545-15550.

4. Newman AM, Liu CL, Green MR, Gentles AJ, Feng W, Xu Y, et al. Robust enumeration of cell subsets from tissue expression profiles. Nat Methods. 2015;12(5):453-457.

5. Charoentong P, Finotello F, Angelova M, Mayer C, Efremova M, Rieder D, et al. Pan-cancer Immunogenomic Analyses Reveal Genotype-Immunophenotype Relationships and Predictors of Response to Checkpoint Blockade. Cell Rep. 2017;18(1):248-262.

6. Li T, Fan J, Wang B, Traugh N, Chen Q, Liu JS, et al. TIMER: A Web Server for Comprehensive Analysis of Tumor-Infiltrating Immune Cells. Cancer Res. 2017;77(21):e108-e110.

7. Jiang P, Gu S, Pan D, Fu J, Sahu A, Hu X, et al. Signatures of T cell dysfunction and exclusion predict cancer immunotherapy response. Nat Med. 2018;24(10):1550-1558.

8. Hoshida Y, Brunet JP, Tamayo P, Golub TR, Mesirov JP. Subclass mapping: identifying common subtypes in independent disease data sets. PLoS One. 2007;2(11):e1195.

9. Hugo W, Zaretsky JM, Sun L, Song C, Moreno BH, Hu-Lieskovan S, et al. Genomic and Transcriptomic Features of Response to Anti-PD-1 Therapy in Metastatic Melanoma. Cell. 2016;165(1):35-44.

10. Chalmers ZR, Connelly CF, Fabrizio D, Gay L, Ali SM, Ennis R, et al. Analysis of 100,000 human cancer genomes reveals the landscape of tumor mutational burden. Genome Med. 2017;9(1):34.

11. Yu H, Li Z, Wang M. Expression and prognostic role of E2F transcription factors in high-grade glioma. CNS Neurosci Ther. 2020;26(7):741-753.
